# Lipopolysaccharide-induced DNA damage response activates DNA-PKcs to drive actin cytoskeleton disruption and cardiac microvascular dysfunction in endotoxemia

**DOI:** 10.7150/thno.111266

**Published:** 2025-04-28

**Authors:** Ying Tan, Yue Ouyang, Lushan Xiao, Jianming Huang, Fuye Li, Zisheng Ma, Chuhong Tan, Weibin Feng, Erica Davis, Yaoping Tang, Xing Chang, Haixia Li

**Affiliations:** 1 Department of Critical Care Medicine, Nanfang Hospital, Southern Medical University, Guangzhou 510515, China.; 2 Department of Critical Care Medicine, The First School of Clinical Medicine, Southern Medical University, Guangzhou 510515, China.; 3 Department of Infectious Diseases, Nanfang Hospital, Southern Medical University, Guangzhou 510515, China.; 4 School of Pharmacy, University of Phoenix, 4035 S Riverpoint Pkwy, Phoenix, AZ 85040, United States.; 5 Faculty of International Education Guangxi University of Chinese Medicine, Nanning 530001, China.; 6 Guang'anmen Hospital of Chinese Academy of Traditional Chinese Medicine, Beijing, China.

**Keywords:** DNA-PKcs, mitochondria, actin, cytoskeleton, DNA damage response

## Abstract

**Rationale:** Sepsis-induced cardiomyopathy is characterized by microvascular injury, which is linked to lipopolysaccharide (LPS)-induced DNA damage response (DDR). This study investigates the role of DNA-PKcs, a key enzyme in the DDR pathway, in driving actin disruption and microvascular dysfunction following LPS exposure.

**Methods:** We analyzed diverse transcriptomic datasets from septic human and murine models using bioinformatics tools to assess DDR pathway activation, correlations, and prognosis. *In vivo*, LPS-challenged mice were treated with inhibitors of DNA-PKcs or mitochondrial fission, and we evaluated cardiac function, microvascular integrity, mitochondrial status, and actin polymerization.

**Results:** Bioinformatic analyses consistently revealed significant activation of the DDR pathway and upregulation of key genes across diverse septic models. Notably, elevated DDR pathway activity was significantly correlated with poor 28-day survival in human sepsis patients. Single-cell analysis localized this DDR gene upregulation predominantly to cardiac endothelial cells (ECs), fibroblasts, and macrophages during sepsis. Within septic capillary ECs, DDR pathway activity scores strongly correlated spatially and functionally with heightened mitochondrial fission and cytoskeletal remodeling pathway activities. *In vivo* experiments confirmed that LPS induced severe systolic and diastolic dysfunction, microvascular damage, and mitochondrial fragmentation, as well as significant actin depolymerization. Inhibition of DNA-PKcs with NU7441 markedly attenuated all these LPS-induced pathologies, improving cardiac function, preserving microvascular structure, preventing mitochondrial fragmentation, and normalizing related gene expression and actin cytoskeleton stability. Additionally, inhibiting mitochondrial fission with Mdivi-1 significantly ameliorated LPS-induced cardiac dysfunction and microvascular injury.

**Conclusions:** Our findings suggest that LPS triggers a DNA-PKcs-dependent DDR that promotes mitochondrial fragmentation and actin disruption, particularly in cardiac ECs, contributing to sepsis-induced cardiomyopathy. Targeting DNA-PKcs or mitochondrial fission may hold therapeutic potential for the treatment of sepsis-induced cardiomyopathy.

## Introduction

Lipopolysaccharide (LPS), found in the cell walls of Gram-negative bacteria, can activate DNA-dependent protein kinase (DNA-PK), a crucial enzyme in DNA repair 1. This activation triggers a DNA damage response, leading to disruptions in the cell's cytoskeleton and ultimately causing damage to the heart's microvasculature. DNA-PK is a pivotal player in the body's response to DNA damage, ensuring the stability of the genome 2. It's a serine/threonine protein kinase composed of Ku proteins and a catalytic subunit, DNA-PKcs 2. DNA-PKcs is activated by DNA double-strand breaks and plays a crucial role in the non-homologous end joining (NHEJ) pathway, one of the major pathways for repairing these breaks 3. DNA-PKcs is recruited to the site of damage by Ku, where it facilitates the movement of Ku into the DNA duplex, allowing DNA-PKcs to act as a tether for the broken DNA ends 2. Upon activation, DNA-PK phosphorylates various proteins involved in DNA repair, including itself and other damage-responsive proteins 4.

The cytoskeleton, a network of protein filaments providing structural support to cells, is disrupted by DNA damage through various mechanisms 5, 6. For example, DNA damage can activate p53, a protein that can induce cell cycle arrest, senescence, and apoptosis. p53 can also directly affect the cytoskeleton by regulating the expression of cytoskeletal proteins or by interacting with cytoskeletal components 7. Oxidative stress, an imbalance between the production of reactive oxygen species and the ability of cells to detoxify them, is a key factor in cardiovascular diseases 8, 9. Oxidative DNA damage can induce changes in the neuronal cell cycle and activate a DNA damage response to promote repair 8, 10. However, under chronic oxidative stress, these processes may be altered, leading to the accumulation of unrepaired DNA damage and continued activation of a DNA damage response, ultimately contributing to neuronal dysfunction and neurodegeneration 8, 11.

Endothelial cells, which line the inner surface of blood vessels, play a critical role in regulating vascular tone, permeability, and inflammation. The cytoskeleton is essential for maintaining the integrity of the endothelial barrier 12. Cytoskeleton dysregulation can disrupt endothelial cell-cell and cell-matrix adhesions, leading to increased endothelial permeability and inflammation 13, 14. This can result in the leakage of fluid and macromolecules from the blood vessels into the surrounding tissues, leading to edema and tissue damage 15, 16. Endothelial cells are constantly exposed to various mechanical forces, including shear stress and circumferential stress 17. These forces play a crucial role in maintaining vascular integrity and regulating endothelial cell function 17. Shear stress, for example, promotes endothelial homeostasis and vascular health 17. Disruption of these mechanical forces, along with cytoskeleton dysregulation, can contribute to endothelial damage 17, 18.

The cardiac microvasculature is a network of small blood vessels that supply blood to the heart muscle 19-21. Endothelial cells in the cardiac microvasculature play a critical role in regulating blood flow and oxygen delivery to the heart. Endothelial damage can lead to impaired coronary microvascular function, resulting in reduced myocardial blood flow and oxygen supply 22-24. This can contribute to various cardiovascular diseases, including myocardial infarction and heart failure 25-27. Microvascular endothelial dysfunction, characterized by impaired or lost homeostatic mechanisms, is an early event in the development of cardiovascular diseases and associated organ damage 28-31.

In the present study, we used the bioinformatic analysis to show that LPS triggers a cascade of events starting with DNA-PK activation, leading to DNA damage response, cytoskeleton dysregulation, and ultimately, cardiac microvascular damage. This intricate pathway highlights the crucial role of DNA-PK in maintaining cellular integrity and the significant impact of endothelial dysfunction in cardiovascular diseases.

## Methods

### Mice

All studies involving animals strictly adhered to Southern Medical University guidelines and received prior clearance from the institution's Institutional Animal Care and Use Committee (IACUC) (No. GSE262911). We utilized male C57BL/6 mice, 8-10 weeks old and weighing 20-25 g. Following a minimum one-week acclimatization period, the mice underwent random assignment into either a control cohort or an experimental sepsis cohort, with six animals designated per group. To establish a model of sepsis-related cardiac dysfunction, animals in the sepsis cohort received a single intraperitoneal (i.p.) administration of *Escherichia coli* lipopolysaccharide (LPS, serotype O111:B4, obtained from Sigma-Aldrich, St. Louis, MO, USA) 32. The LPS was given at a dosage of 10 mg/kg, prepared in sterile phosphate-buffered saline (PBS) devoid of endotoxins. Animals assigned to the control cohort were administered a matched volume of the sterile PBS vehicle via the same i.p. injection route. Functional assessments, such as echocardiography, along with terminal harvesting of tissues for histological, ultrastructural, and molecular investigations, were carried out 24 h following the administration of either LPS or PBS vehicle 29, 31.

### Ultrasound assessment of cardiac function

Following the LPS challenge, ultrasound cardiac evaluations were conducted utilizing the Vevo 2100 platform (VisualSonics, Toronto, Canada), adhering to established methodologies. M-mode imaging acquired from both long- and short-axis views was employed to quantify ventricular chamber dimensions and myocardial wall thickness during systolic and diastolic phases. Parameters including left ventricular (LV) internal diameter at end-diastole (LVDd) and end-systole (LVSd), along with calculated LV volumes at end-diastole (LVEDV) and end-systole (LVESV), served as indicators reflecting potential chamber dilation. The estimated LV mass offered an index related to myocardial hypertrophic changes. To gauge contractile performance, the left ventricular ejection fraction (LVEF) was derived via the standard formula: ([LVEDV - LVESV] / LVEDV) × 100. Assessments of blood flow and myocardial velocity using Doppler techniques were obtained from an apical four-chamber perspective to analyze LV filling patterns and diastolic characteristics. The speed of blood entering the LV across the mitral valve was quantified through pulsed-wave Doppler interrogation 33, 34. Key recorded metrics comprised the peak early (E) and late (A) diastolic filling velocities, plus the derived E/A ratio. Myocardial tissue speeds via Doppler were captured by positioning a 1.0 mm sample volume at the medial segment of the mitral annulus. Subsequent analysis yielded the early (E′) and late (A′) diastolic tissue velocities. Ratios combining blood flow and tissue motion, specifically the mitral E/E′ ratio and the tissue Doppler E′/A′ ratio, were computed to evaluate diastolic properties. All Doppler spectral data were captured over three to five consecutive heartbeats employing a sweep display rate of 100 mm/s. The preset for color flow imaging established a Nyquist limit of 0.44 m/s. Analysis of the acquired echocardiographic images was performed offline by an operator who remained unaware of the group allocation for each animal 35, 36.

### Tissue processing, histology, and immunohistochemistry

Following humane euthanasia, animals underwent systemic perfusion via the left ventricle, initially with saline solution, followed by fixation using 4% paraformaldehyde (PFA) in PBS for 15 min. The aorta and carotid arteries were subsequently dissected, embedded within optimal cutting temperature (OCT) compound, and immediately cryopreserved. Serial frozen sections, each 7 µm thick, were prepared utilizing a cryostat. For morphological assessment, Hematoxylin and Eosin (HE) staining was conducted with a commercial kit (Solarbio, Cat. No. G1346), following the provided supplier protocol, to examine microvascular structure. Microscopic images were acquired using a Leica imaging system; subsequent quantitative evaluations employed Image-Pro Plus software (Media Cybernetics, USA) 37, 38.

For immunohistochemical analysis of eNOS on these cryosections, antigen unmasking was first performed using a citrate-based retrieval solution heated near boiling for 25 min 39, 40. Sections were then subjected to a 30 min blocking step with 5% bovine serum albumin (BSA). Incubation with the primary antibody targeting eNOS proceeded overnight at 4°C. Subsequently, sections were incubated with an HRP-conjugated secondary antibody for 1 h at 37°C. Antibody binding was visualized using a 3,3'-diaminobenzidine (DAB) substrate kit (Gene Tech, Shanghai, China), followed by nuclear counterstaining with hematoxylin. Images for IHC analysis were captured on a standard Olympus microscope (Tokyo, Japan) 41, 42.

Quantification of the eNOS immunostaining utilized digital image analysis. The Image-Pro Plus software (version 6.0; Media Cybernetics, Inc.) facilitated this analysis of antigen expression levels. The total positively stained area within each field was measured in pixels, providing integrated optical density (IOD) values as an outcome 43, 44. As an alternative quantification approach, the histological score (H-score) method was also applied. This H-score calculation considered both the percentage of cells positive for the target protein and the intensity of the staining observed. Staining intensity received a semi-quantitative rating: 0 for negative, 1 for weak, 2 for moderate, or 3 for strong 45, 46. The final H-score resulted from multiplying the positive cell percentage by the assigned intensity rating. Samples exhibiting an H-score exceeding the calculated average were classified as having high expression, whereas those below the average indicated low expression. All scoring evaluations were conducted independently by two pathologists who were kept unaware of the experimental group assignments for the samples 21, 47.

### Gene expression analysis via qRT-PCR

Overall RNA isolation was achieved using RNAiso Plus reagent (Takara, #9109), adhering strictly to the provided supplier protocol 48, 49. Following isolation, complementary DNA (cDNA) synthesis occurred employing the RT reagent Kit that includes a gDNA Eraser step (Takara, #RR047). Real-time quantitative PCR analyses were conducted in triplicate reactions utilizing a LightCycler 480 system (Roche Diagnostics, Germany) 50, 51. Expression findings were adjusted based on the transcript levels of the internal reference gene, GAPDH 52, 53. Calculation of relative expression employed the comparative Ct (ΔΔCt) methodology. The oligonucleotide primer sequences utilized for amplification are detailed below: Fis1, F, 5′- GCTGGTTCTGTGTCCAAGAGCA-3′, R, 5′-GACATAGTCCCGCTGTTCCTCT-3′; Drp1, F, 5′- GCGAACCTTAGAATCTGTGGACC-3′, R, 5′-CAGGCACAAATAAAGCAGGACGG-3′; Mff, F, 5′- GAGTGGTGGAGATGGTGGAA-3′, R, 5′-GGTTGACACAGAGTGGGAAA-3′; Mfn2, F, 5′-GTGGAATACGCCAGTGAGAAGC-3′; R, 5′-CAACTTGCTGGCACAGATGAGC-3′; Mfn1, F, 5′-CCAGGTACAGATGTCACCACAG-3′, R, 5′-TTGGAGAGCCGCTCATTCACCT-3′.

### Cellular immunofluorescence staining

Initially, cellular specimens were cultured directly onto coverslips 54, 55. These were subsequently preserved using a 4% paraformaldehyde solution for a 30 min fixation period. Following fixation, membrane permeabilization was achieved with a 15 min treatment of 0.5% Triton X-100. A solution containing 5% bovine serum albumin (BSA) was then applied to obstruct potential non-specific antibody binding areas 56, 57. The prepared specimens were exposed to the designated primary antibodies, allowing incubation to proceed overnight under refrigeration at 4°C. After completing five washing sequences with TBST buffer, samples underwent incubation with secondary antibodies. These secondary reagents were conjugated to either Alexa Fluor 488 or Alexa Fluor 594 (obtained from Abcam) 58, 59. Subsequent to this 1 h secondary antibody step, the coverslips received washing cycles using PBS, performed while protected from light. Nuclear material was counterstained employing 4′,6-diamidino-2-phenylindole (DAPI, sourced from Beyotime, Shanghai, China). Finally, fluorescent images were acquired utilizing a laser scanning confocal microscope (Olympus LV3000 model, Tokyo, Japan) 60, 61.

### Computational processing of single-cell RNA sequencing data

Regarding the examination of the GSE190856 dataset, relevant information was consolidated to facilitate subsequent investigations 62, 63. To summarize, the initial unique molecular identifier (UMI) count matrices underwent processing utilizing the Seurat software package. The following quality control standards were implemented for each sample: cellular units exhibiting minimal library content (fewer than 500 counts per unit) were omitted, along with genetic features detected in under three cells within any given sample 64, 65. Numerical counts were adjusted on a per-cell basis, transformed into a logarithmic representation, and then amplified by a scaling factor of 10,000. Features demonstrating substantial variability were pinpointed by employing Seurat's FindVariableFeatures function, selecting 2,000 such features 66, 67. Subsequently, this dataset underwent scaling via the ScaleData routine. A reduction in dimensionality was accomplished using the RunPCA function, focusing on the top 30 principal components. The resultant gene expression matrices were merged into a singular dataset employing the Harmony integration method. Following this, a dimensionality reduction visualization was produced with RunUMAP, utilizing the initial 30 dimensions 68, 69. Finally, distinct cell populations were assigned identities founded on recognized canonical gene markers.

### Gene co-expression network construction

We implemented Weighted Gene Co-expression Network Analysis (WGCNA) utilizing R software version 4.0.3 equipped with the WGCNA library, version 1.69. A signed-hybrid approach was selected for defining the network topology. The appropriate soft-thresholding power (β identified as 12) was ascertained by evaluating scale-free fit and network modularity according to established methodologies. Furthermore, network modules were delineated specifying a minClusterSize of 30 and deepSplit value of 2; other parameters remained at their default settings 70, 71. Following the identification of co-expression modules, their relationship to phenotypic traits was assessed by correlating module eigengenes with phenotypes using Spearman's method. Visualizations of the network structure were produced employing the R package 'igraph' version 1.2.6 72, 73.

### Reduced dimension analysis, clustering, and differential expression

Identification of unsupervised cell groups and differential gene expression assessment were conducted using the Seurat R package (v4.0.0). Cell partitioning utilized a shared nearest neighbor approach derived from the top 50 principal components, which captured the primary variance within the integrated dataset as indicated by principal component analysis (PCA). The Louvain algorithm, with a resolution setting of 0.7, was applied to define the resultant clusters. These groupings were subsequently refined, potentially merged, and assigned identities relying on canonical marker genes pinpointed via the FindAllMarkers function. Major identified cell types (including cardiomyocytes, endothelial populations, immune components, and others) underwent separate subset clustering for more detailed examination 74, 75. Through the FindAllMarkers utility, marker genes characterizing these subclusters were found, facilitating the manual annotation of distinct myeloid-like subsets 76, 77. Graphical representations, encompassing heatmaps 20, 78, 79, UMAP plots, violin diagrams, feature expression maps, and dot plots, were generated utilizing Seurat in conjunction with ggplot2 (v3.3.5) and pheatmap (v1.0.12). Differential expression testing within Seurat employed the Wilcoxon Rank Sum test; analyses considered genes expressed in a minimum threshold of 25% of cells within a relevant group. Outcomes were visualized using built-in Seurat capabilities or the VennDiagram package (v1.7.3) 80, 81.

### Assessment of abundance differences

We conducted an analysis of abundance variations focusing on the myeloid cell subsets derived from different experimental conditions. This process utilized the miloR package (version 1.2.0) for constructing a k-Nearest Neighbor graph (parameters: k=30, d=30) and subsequently defining cellular neighborhoods with a proportion setting of 0.2 77, 82. Cell counts within these neighborhoods were determined using the countCells function, following the calculation of distances with calcNhoodDistance based on 30 dimensions 43, 83. Variations in abundance across the specified conditions were statistically evaluated employing the testNhoods function. Results were then visualized using functionalities including buildNhoodGraph and plotDAbeeswarm, applying significance thresholds (alpha) set at either 0.05 or 0.5 84, 85.

### Functional pathway investigation

Single-sample gene set enrichment analysis (ssGSEA) computations were executed utilizing the R package GSVA (version 1.42.0) 76, 86. This involved importing Hallmark, KEGG, Reactome, and Gene Ontology (GO) pathway definitions from the Molecular Signatures Database (MSigDB version 7.4), a step facilitated by the GSEABase R library (version 1.56.0). Following score calculation, the Limma package (version 3.50.0) was employed to pinpoint gene sets demonstrating statistically significant enrichment differences based on these computed pathway scores 45, 87. Visualization of results was accomplished using ggplot2 for various plots, and pheatmap was specifically used to display heatmaps reflecting the normalized average pathway scores within each analyzed subset 88, 89.

### Data analyses

All procedures were replicated independently on at least three separate occasions. Each individual experimental run incorporated three biological replicates. The precise quantity of independent repetitions is detailed within the respective figure captions. Numerical findings are generally shown as the average ± standard deviation (SD), calculated relative to the control treatment (defined as 100%) unless noted differently within a figure's description. For statistical evaluation, the two-tailed Mann-Whitney U test was employed; results were deemed significant if p < 0.05 (#). Significance levels meeting these thresholds are denoted by asterisks either directly on the graphs or within the accompanying figure descriptions. Graphical data presentations were created using GraphPad Prism 9 software, while diagrams were constructed with Biorender.

## Results

### LPS induces a robust DNA damage response in human PBMCs

To investigate the effects of LPS on the DNA damage response (DDR) in cardiac microvascular endothelial cells, we analyzed RNA sequencing data from the GSE262911 dataset, which examined human CD14^+^ monocytes stimulated with LPS. Specifically, the dataset (GSM8181391) includes CD14^+^ monocytes isolated from peripheral blood mononuclear cells (PBMCs) of healthy donors. These monocytes were cultured in the presence of M-CSF (20 ng/mL) and pre-treated with IFNγ (100 Units/mL) overnight, followed by stimulation with LPS (10 ng/mL) for 3 h. RNA was then extracted, and polyA-selected RNA sequencing was performed using the Illumina NovaSeq 6000 platform. Reads were aligned to the human genome (hg38) and quantified using STAR aligner against Gencode v38 annotations.

**Figure 1:**
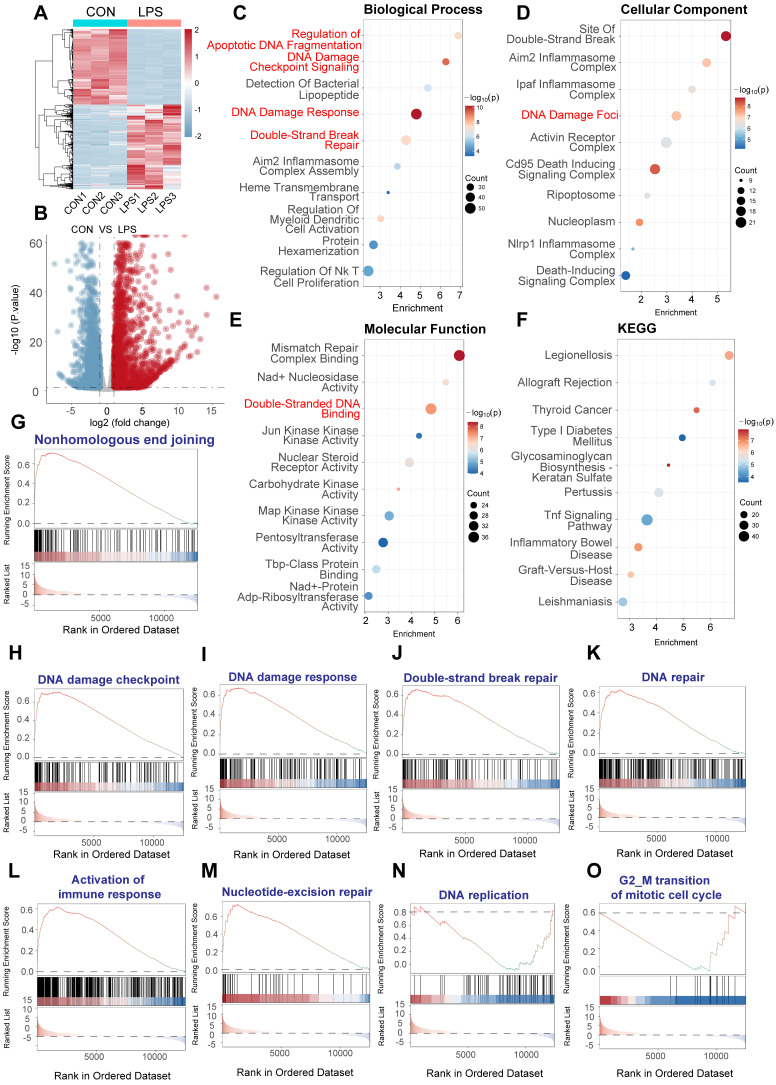
** LPS induces DNA damage response (DDR) activation in human CD14^+^ monocytes.** RNA sequencing data from human CD14^+^ monocytes, pre-treated with IFNγ (100 U/mL) overnight and then stimulated with LPS (10 ng/mL) for 3 h, were analyzed (GSE262911 dataset, GSM8181391). Reads were aligned to the human genome (hg38) and quantified using STAR aligner against Gencode v38 annotations. (A) Heatmap of differentially expressed genes (DEGs) between control and LPS-treated monocytes. Color scale represents gene expression levels (red: upregulation, blue: downregulation). (B) Volcano plot of DEGs. Statistical significance is plotted against the magnitude of change. Significantly upregulated genes are in red, and downregulated genes are in blue. (C) Gene Ontology (GO) enrichment analysis of biological processes. Dot size represents gene count. Key terms related to DNA damage and repair are highlighted. (D) GO enrichment analysis of cellular components. Dot size and color as in (C). Locations relevant to DDR are emphasized. (E) GO enrichment analysis of molecular functions. Dot size and color as in (C). Activities related to DNA binding, repair, and enzymatic processes are highlighted. (F) KEGG pathway enrichment analysis. Dot size and color as in (C). Pathways related to inflammation, DNA damage, and disease are noted. (G-O) Gene Set Enrichment Analysis (GSEA) plots. Enrichment scores (ES) are plotted against the ranked gene list. The running ES is shown as a blue line; vertical black lines indicate gene positions within the gene set. Positive ES indicates upregulation in the LPS-treated group. Gene sets analyzed: (G) Nonhomologous end joining. (H) DNA damage checkpoint. (I) DNA damage response. (J) Double-strand break repair. (K) DNA repair. (L) Activation of immune response. (M) Nucleotide-excision repair. (N) DNA replication. (O) G2/M transition of mitotic cell cycle.

We compared the gene expression profiles of these LPS-treated CD14^+^ monocytes to untreated controls within the GSE262911 dataset. Unsupervised hierarchical clustering of differentially expressed genes (DEGs) revealed a clear separation between LPS-treated and control samples, indicating a substantial alteration in the transcriptome (Figure 1A). A volcano plot analysis further highlighted the magnitude and statistical significance of these changes, demonstrating a large number of genes significantly upregulated and downregulated upon LPS stimulation (Figure 1B; adjusted P-value < 0.05, |log_2_(fold change)| > 1).

To gain insights into the biological functions and pathways affected by LPS, we performed Gene Ontology (GO) enrichment analysis on the DEGs. This analysis revealed a striking enrichment of terms related to the DNA damage response, including "DNA damage response," "double-strand break repair," and "apoptotic DNA fragmentation" (Figure 1C, Biological Process). Consistent with this, GO analysis of cellular components highlighted the enrichment of "site of double-strand break" and "DNA damage foci," among other relevant locations (Figure 1D). Furthermore, GO analysis of molecular functions showed significant enrichment of terms such as "double-stranded DNA binding" and activities related to various kinases involved in DNA repair (Figure 1E). KEGG pathway enrichment analysis further corroborated these findings, with significant enrichment of pathways related to DNA repair mechanisms, as well as pathways involved in immune and inflammatory response (Figure 1F).

To further validate and refine these observations, we performed Gene Set Enrichment Analysis (GSEA) using predefined gene sets related to specific aspects of DNA damage and repair. GSEA confirmed the significant upregulation of gene sets associated with "nonhomologous end joining" (Figure 1G), "DNA damage checkpoint" (Figure 1H), "DNA damage response" (Figure 1I), "double-strand break repair" (Figure 1J), and "DNA repair" in general (Figure 1K) in LPS-treated PBMCs. We also observed the enrichment of "Activation of immune response" (figure 1L), which provided a link between DNA damage and immune. Moreover, GSEA showed the upregulation for gene sets involved in "nucleotide-excision repair" (Figure 1M), "DNA replication" (Figure 1N), and "G2/M transition of mitotic cell cycle" (Figure 1O), indicating a comprehensive activation of DNA repair and cell cycle regulatory mechanisms.

In summary, these data provide strong evidence that LPS stimulation induces a robust transcriptional response characterized by the activation of multiple DNA damage response pathways, encompassing DNA damage detection, checkpoint signaling, and various DNA repair mechanisms. The simultaneous upregulation of immune related pathways underscores the intricate interplay between inflammation and genotoxic stress induced by LPS.

### LPS-induced sepsis elicits a robust DNA damage response in murine PBMCs

**Figure 2:**
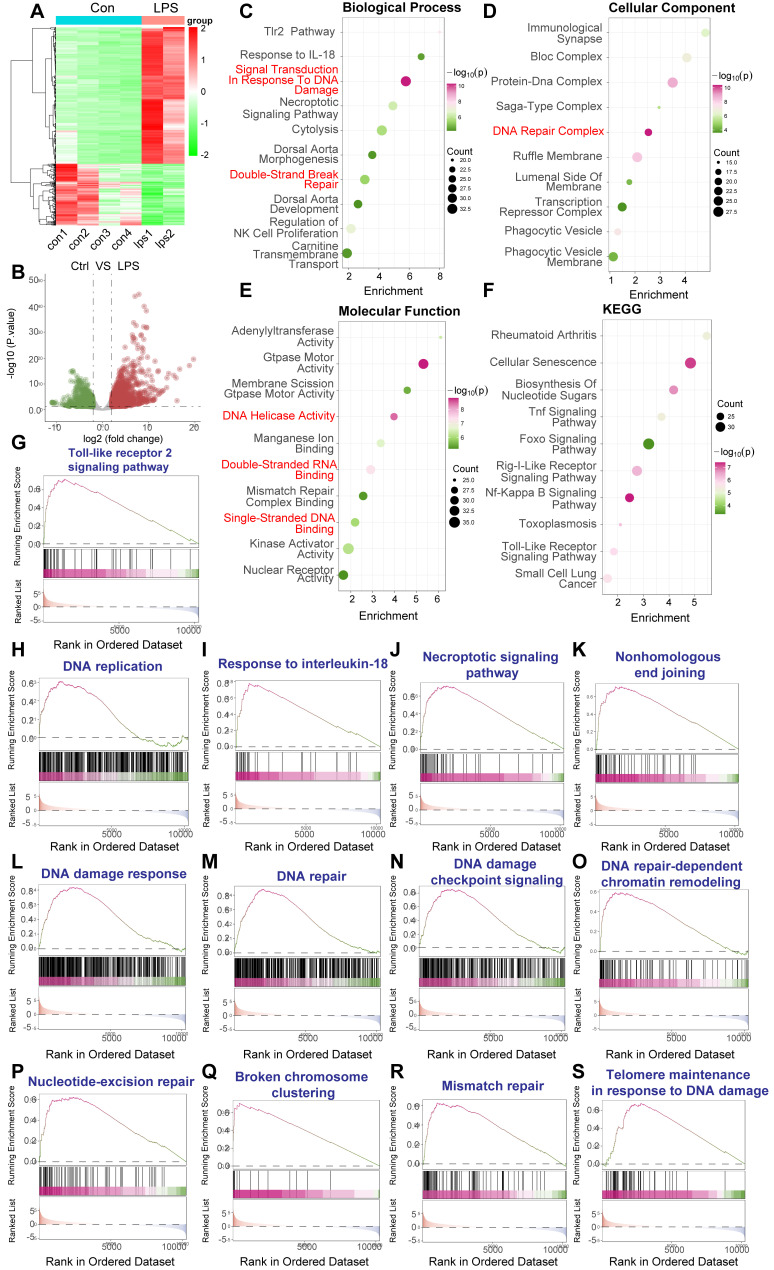
** LPS-induced sepsis activates DNA damage response and inflammatory pathways in murine PBMCs.** Bioinformatic analysis of RNA sequencing data from peripheral blood mononuclear cells (PBMCs) isolated from a murine model of LPS-induced sepsis. Data were generated using Illumina sequencing platforms. (A) Heatmap illustrating differentially expressed genes (DEGs) between control (Con) and LPS-treated mice PBMCs, showing distinct clustering based on treatment group. Color scale represents Z-score normalized expression levels (Red: higher expression, Blue: lower expression). (B) Volcano plot depicting the magnitude of gene expression change versus statistical significance. Red dots indicate significantly upregulated genes (n=1062); blue dots indicate significantly downregulated genes (n=479). Thresholds for significance were set at adjusted. (C-F) Functional enrichment analyses of DEGs. Dot size represents the number of genes enriched in the term/pathway; color intensity corresponds to the statistical significance. (C) Gene Ontology (GO) Biological Process enrichment, highlighting terms related to DNA damage response, DNA repair, and immune/inflammatory signaling. (D) GO Cellular Component enrichment, indicating involvement of structures like the DNA Repair Complex. (E) GO Molecular Function enrichment, showing enrichment of activities such as DNA Helicase Activity and Mismatch Repair Complex Binding. (F) Kyoto Encyclopedia of Genes and Genomes (KEGG) pathway enrichment analysis, revealing significant enrichment in inflammatory pathways and disease-related pathways. (G-S) Gene Set Enrichment Analysis (GSEA) plots for selected gene sets. The plots show the running enrichment score (ES, blue line) across the ranked list of genes. Vertical black lines indicate the positions of genes within the specific gene set. Positive ES indicates enrichment (upregulation) of the gene set in the LPS-treated group. Gene sets shown are relevant to inflammation, cell death, and various aspects of DNA damage response and repair.

To investigate the *in vivo* relevance of DNA damage response (DDR) activation in sepsis, we analyzed publicly available RNA sequencing data from peripheral blood mononuclear cells (PBMCs) isolated from a murine model of LPS-induced sepsis (GEO accession: GSE224146). Comparing the transcriptomes of PBMCs from LPS-treated mice versus control mice, unsupervised hierarchical clustering revealed distinct gene expression profiles separating the two groups (Figure 2A). Volcano plot analysis identified a substantial number of differentially expressed genes (DEGs); specifically, 1062 genes were significantly upregulated and 479 genes were significantly downregulated in the sepsis model compared to controls, using thresholds of adjusted P-value < 0.05 and |log_2_(fold change)| > 1 (Figure 2B).

To elucidate the biological implications of these transcriptional changes, we performed functional enrichment analyses. Gene Ontology (GO) analysis of biological processes showed significant enrichment of terms associated with immune and inflammatory responses, such as "Tlr2 Pathway" and "Response to IL-18", alongside key DDR-related terms including "Signal Transduction In Response To DNA Damage" and "Double Strand Break Repair" (Figure 2C). Consistent with DDR activation, GO cellular component analysis highlighted enrichment in "DNA Repair Complex" (Figure 2D). Furthermore, GO molecular function analysis revealed enrichment in activities crucial for DNA metabolism and repair, such as "DNA Helicase Activity", "Mismatch Repair Complex Binding", and "Single-Stranded DNA Binding" (Figure 2E). KEGG pathway analysis corroborated these findings, demonstrating enrichment in inflammatory pathways like "Toll-Like Receptor Signaling Pathway" and "NF-kappa B Signaling Pathway," alongside pathways related to cellular stress and disease (Figure 2F).

To further probe the specific pathways involved, we conducted Gene Set Enrichment Analysis (GSEA). This analysis confirmed the significant upregulation of inflammatory pathways, including the "Toll-like receptor 2 signaling pathway" (Figure 2G) and "Response to interleukin-18" (Figure 2I), as well as pathways linked to cell stress and death like the "Necroptotic signaling pathway" (Figure 2J). Crucially, GSEA demonstrated strong, significant enrichment of multiple gene sets directly involved in the DNA damage response and repair, encompassing "Nonhomologous end joining" (Figure 2K), "DNA damage response" (Figure 2L), "DNA repair" (Figure 2M), "DNA damage checkpoint signaling" (Figure 2N), "DNA repair-dependent chromatin remodeling" (Figure 2O), "Nucleotide-excision repair" (Figure 2P), "Broken chromosome clustering" (Figure 2Q), "Mismatch repair" (Figure 2R), and "Telomere maintenance in response to DNA damage" (Figure 2S). Enrichment was also noted for "DNA replication" (Figure 2H), suggesting potential cell cycle perturbations alongside DNA damage.

Collectively, these bioinformatic analyses of the GSE224146 dataset provide robust *in vivo* evidence that LPS-induced sepsis in a murine model triggers significant activation of both inflammatory signaling and comprehensive DNA damage response pathways within PBMCs. This supports the hypothesis that genotoxic stress is a relevant component of the systemic response to sepsis and may contribute to sepsis-associated pathologies.

### DNA damage response pathways are activated in PBMCs from human septic shock patients

To further validate the activation of the DNA Damage Response (DDR) in a clinically relevant human setting, we analyzed a publicly available microarray dataset (GEO accession: GSE131761). This dataset compared gene expression profiles in peripheral blood mononuclear cells (PBMCs) derived from post-surgical patients experiencing septic shock versus those experiencing non-septic shock. The expression profiling was performed using the Agilent Whole Human Genome Microarray 4x44K v2 platform (GPL13497).

**Figure 3:**
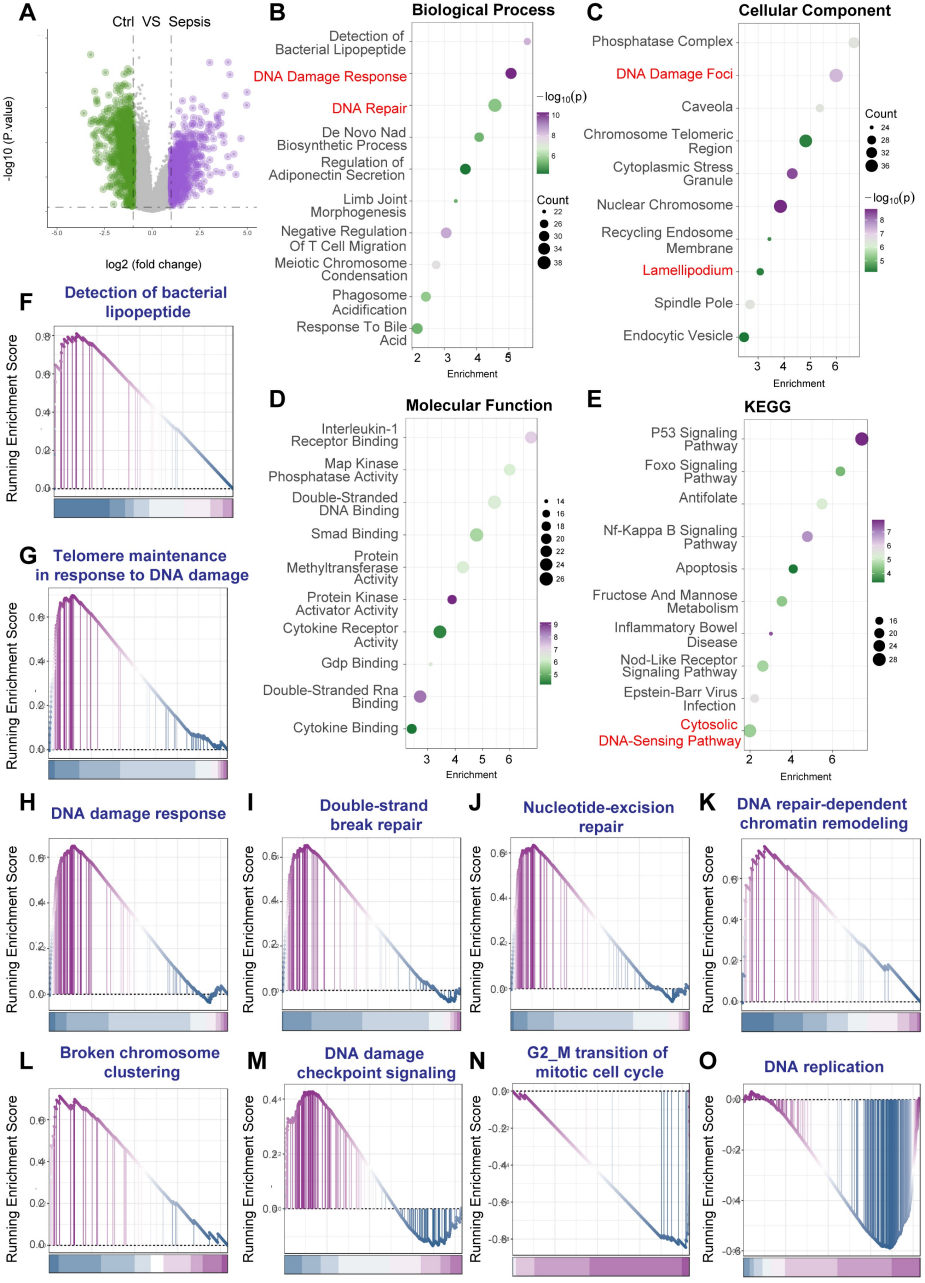
** Activation of DNA damage response pathways in peripheral blood mononuclear cells from human septic shock patients.** Bioinformatic analysis of microarray gene expression data from peripheral blood mononuclear cells (PBMCs) comparing post-surgical patients diagnosed with septic shock (Sepsis) versus post-surgical patients with non-septic shock (Ctrl) (*Homo sapiens*; GEO accession: GSE131761). Gene expression was profiled using the Agilent Whole Human Genome Microarray platform (GPL13497). (A) Volcano plot illustrating differential gene expression between septic shock and non-septic shock PBMCs. (B-E) Functional enrichment analyses performed on the identified DEGs. Dot plots display significantly enriched terms for (B) Gene Ontology (GO) Biological Process (BP), (C) GO Cellular Component (CC), (D) GO Molecular Function (MF), and (E) Kyoto Encyclopedia of Genes and Genomes (KEGG) pathways. Within these plots, dot size typically corresponds to the number of genes associated with the term, and color intensity reflects the statistical significance. Key enriched terms highlighted include those related to DNA Damage Response, DNA Repair, DNA Damage Foci, Double-Stranded DNA Binding, p53 Signaling, and inflammatory pathways. (F-O) Gene Set Enrichment Analysis (GSEA) plots showing the enrichment profiles for selected gene sets when comparing septic shock versus non-septic shock PBMCs. The plots display the running Enrichment Score (ES) across the ranked gene list, with vertical lines indicating the positions of genes within the set. A positive ES indicates significant enrichment (upregulation) of the gene set in the septic shock group.

Differential gene expression analysis between septic shock and non-septic shock PBMCs revealed numerous significantly altered transcripts (Figure 3A). To elucidate the biological implications of these DEGs, functional enrichment analyses were performed. Gene Ontology (GO) analysis demonstrated significant enrichment for Biological Process terms related to genotoxic stress, including "DNA Damage Response" and "DNA Repair", alongside terms reflecting innate immune activation such as "Detection of bacterial lipopeptide" (Figure 3B). Analysis of Cellular Component revealed enrichment in relevant locations such as "DNA Damage Foci" and "Chromosome Telomeric Region" (Figure 3C). Enriched Molecular Function terms included activities like "Double-Stranded DNA Binding" and "Protein Kinase Activator Activity" (Figure 3D). Furthermore, Kyoto Encyclopedia of Genes and Genomes (KEGG) pathway analysis identified significant enrichment in key pathways central to cellular stress responses and inflammation, including the "p53 Signaling Pathway", "NF-kappa B Signaling Pathway", "Apoptosis", and the "Cytosolic DNA-Sensing Pathway" (Figure 3E).

To further substantiate the activation status of specific pathways, Gene Set Enrichment Analysis (GSEA) was employed. GSEA showed significant positive enrichment for gene sets related to innate immunity ("Detection of bacterial lipopeptide", Figure 3F). Crucially, multiple gene sets intrinsically linked to DNA damage recognition, signaling, and various repair mechanisms exhibited significant enrichment in the septic shock group. These included "Telomere maintenance in response to DNA damage" (Figure 3G), "DNA damage response" (Figure 3H), "Double-strand break repair" (Figure 3I), "Nucleotide-excision repair" (Figure 3J), "DNA repair-dependent chromatin remodeling" (Figure 3K), and "Broken chromosome clustering" (Figure 3L). Additionally, gene sets associated with cell cycle regulation and DNA replication ("DNA damage checkpoint signalling", Figure 3M; "G2/M transition of mitotic cell cycle", Figure 3N; "DNA replication", Figure 3O) were also significantly enriched.

Collectively, the analysis of the GSE131761 dataset, derived from PBMCs of human post-surgical septic shock patients, provides robust evidence for the activation of comprehensive DNA Damage Response pathways, intertwined with pronounced inflammatory signaling. These findings strongly underscore the clinical relevance of DDR activation within circulating immune cells during the systemic response to human sepsis.

### DNA damage response is activated in human septic cardiomyopathy heart tissue

To directly investigate whether DNA Damage Response (DDR) activation occurs within the cardiac tissue itself during human sepsis, we analyzed the publicly available microarray dataset GSE79962. This dataset includes gene expression profiles from human heart tissue samples. Specifically, it compares tissue obtained from patients who died from sepsis (resulting in septic cardiomyopathy, SCM) to non-failing donor hearts used as controls. The expression profiling was performed using the Affymetrix Human Gene 1.0 ST Array platform (GPL6244).

**Figure 4:**
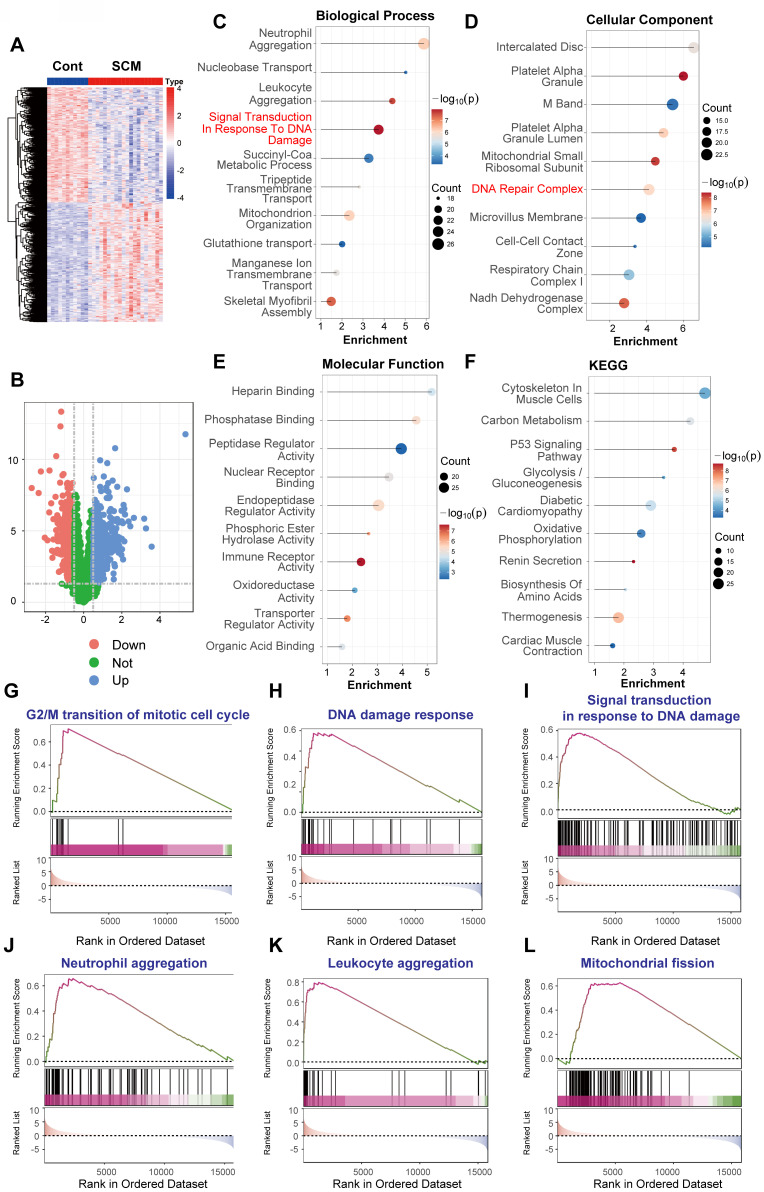
** DNA damage response activation is evident in human septic cardiomyopathy heart tissue.** Bioinformatic analysis of microarray gene expression data from human heart tissue comparing samples obtained from patients who died from sepsis (Septic Cardiomyopathy, SCM) versus non-failing donor hearts (Control, Cont). Expression profiling was performed using the Affymetrix Human Gene 1.0 ST Array platform (GPL6244). (A) Heatmap displaying differentially expressed genes (DEGs) between SCM and Cont heart tissue samples, illustrating distinct clustering based on group. Color scale represents Z-score normalized expression levels (Red: higher expression, Blue: lower expression). (B) Volcano plot visualizing gene expression changes against statistical significance. Red dots indicate significantly upregulated genes (n=459); blue dots indicate significantly downregulated genes (n=442). (C-F) Functional enrichment analyses performed on the DEGs identified in SCM hearts compared to controls. Dot size corresponds to the number of genes enriched in the term/pathway; color intensity reflects the statistical significance. (C) Gene Ontology (GO) Biological Process enrichment. (D) GO Cellular Component enrichment. (E) GO Molecular Function enrichment, indicating alterations in various binding and enzymatic activities. (F) Kyoto Encyclopedia of Genes and Genomes (KEGG) pathway enrichment. (G-L) Gene Set Enrichment Analysis (GSEA) plots for selected functionally relevant gene sets. The plots show the running enrichment score (ES, blue/purple line) across the ranked gene list, with vertical lines marking the positions of genes within the set. A positive ES indicates enrichment (upregulation) of the gene set in the SCM group.

Analysis of this microarray data revealed distinct transcriptional signatures between SCM and control hearts. Unsupervised hierarchical clustering based on differentially expressed genes (DEGs) showed clear separation of the two groups (Figure 4A). Differential expression analysis identified 459 genes as significantly upregulated and 442 genes as significantly downregulated in SCM hearts relative to non-failing controls, applying thresholds of adjusted P-value < 0.05 and |log_2_(fold change)| > 0.5 (Figure 4B).

Functional enrichment analyses were performed on these DEGs derived from the heart tissue microarray data. Gene Ontology (GO) analysis of biological processes indicated significant enrichment of terms related to "Signal Transduction In Response To DNA Damage," alongside inflammatory processes like "Neutrophil Aggregation" and "Leukocyte Aggregation," and alterations in metabolic and transport pathways (Figure 4C). Correspondingly, GO analysis of cellular components showed enrichment in "DNA Repair Complex," as well as mitochondrial components ("Mitochondrial Small Ribosomal Subunit," "Respiratory Chain Complex I") and cardiac structural elements ("Intercalated Disc") (Figure 4D). GO molecular function analysis pointed towards altered enzymatic activities and binding properties (Figure 4E). Kyoto Encyclopedia of Genes and Genomes (KEGG) pathway analysis further implicated DDR, revealing enrichment in the "p53 Signaling Pathway." Additionally, pathways related to cardiac function ("Diabetic Cardiomyopathy," "Cardiac Muscle Contraction") and cellular metabolism ("Oxidative Phosphorylation," "Carbon Metabolism," "Glycolysis / Gluconeogenesis") were significantly enriched (Figure 4F).

To further explore the activation status of specific pathways within the septic heart tissue, Gene Set Enrichment Analysis (GSEA) was conducted. GSEA confirmed significant positive enrichment in the SCM group for gene sets associated with cell cycle regulation potentially linked to DDR ("G2/M transition of mitotic cell cycle," Figure 4G). Crucially, gene sets directly reflecting DDR activation ("DNA damage response," Figure 4H; "Signal transduction in response to DNA damage," Figure 4I) were significantly enriched. Furthermore, GSEA highlighted enrichment in inflammatory pathways ("Neutrophil aggregation," Figure 4J; "Leukocyte aggregation," Figure 4K) and pathways related to cellular stress and mitochondrial dynamics ("Mitochondrial fission," Figure 4L).

In summary, the analysis of gene expression profiles directly from human heart tissue comparing samples from patients who died of sepsis to non-failing donor hearts (GSE79962) provides compelling evidence for the activation of DNA damage response pathways within the myocardium itself during fatal human sepsis. This DDR activation occurs alongside significant inflammatory infiltration signatures and metabolic alterations, strongly suggesting that DNA damage contributes directly to the pathophysiology of cardiac dysfunction in human sepsis.

### DNA damage response is activated in a murine model of septic cardiomyopathy

To further investigate the activation of the DNA Damage Response (DDR) within cardiac tissue in a controlled *in vivo* model, we analyzed RNA sequencing data from the GSE267388 dataset. The data compares heart tissue from C57BL/6 mice subjected to lipopolysaccharide (LPS)-induced sepsis (via a single intraperitoneal injection of 10 mg/kg LPS for 12 h, modeling septic cardiomyopathy, SCM) against heart tissue from vehicle (PBS)-treated control mice (*Mus musculus*).

**Figure 5:**
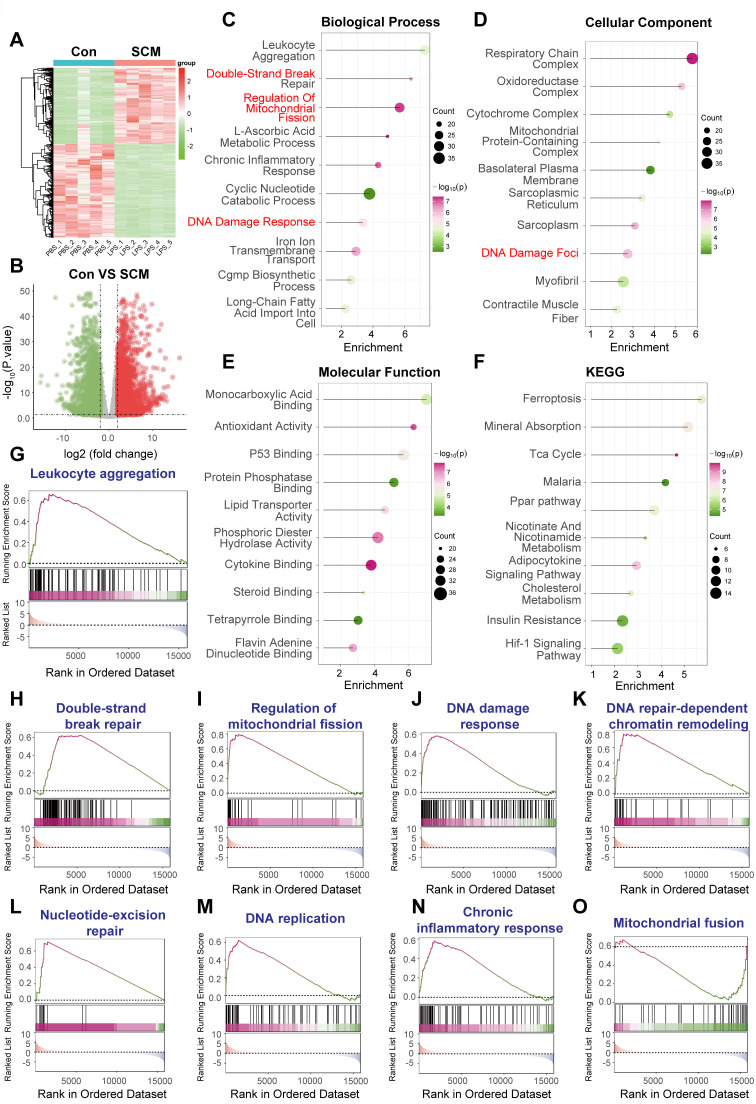
** DNA damage response activation in mouse hearts following LPS-induced septic cardiomyopathy.** Bioinformatic analysis of RNA sequencing data obtained from heart tissue comparing C57BL/6 mice subjected to lipopolysaccharide (LPS)-induced sepsis (modeling Septic Cardiomyopathy, SCM; 10 mg/kg i.p. LPS for 12 h) versus vehicle-treated controls (Con; PBS). (A) Heatmap visualizing differentially expressed genes (DEGs) between SCM and Con mouse hearts, demonstrating distinct clustering based on treatment group. Color scale represents Z-score normalized expression levels (Red: higher expression, Blue: lower expression). (B) Volcano plot displaying gene expression changes against statistical significance. Red dots indicate significantly upregulated genes (n=1558); green dots indicate significantly downregulated genes (n=1909). (C-F) Functional enrichment analyses performed on the DEGs identified in SCM mouse hearts compared to controls. Dot size corresponds to the number of genes enriched in the term/pathway; color intensity reflects the statistical significance. (C) Gene Ontology (GO) Biological Process enrichment. (D) GO Cellular Component enrichment. (E) GO Molecular Function enrichment. (F) Kyoto Encyclopedia of Genes and Genomes (KEGG) pathway analysis. (G-O) Gene Set Enrichment Analysis (GSEA) plots for selected functionally relevant gene sets. The plots show the running enrichment score (ES, colored line) across the ranked gene list, with vertical lines marking the positions of genes within the set. A positive ES indicates enrichment (upregulation) of the gene set in the SCM (LPS-treated) group.

Analysis of this RNA sequencing data revealed significant alterations in cardiac gene expression between the LPS-treated SCM group and the PBS controls. Unsupervised hierarchical clustering based on differentially expressed genes (DEGs) clearly separated the SCM samples from the controls (Figure 5A). Differential expression analysis identified a substantial transcriptional response: 1558 genes were significantly upregulated and 1909 genes were significantly downregulated in the hearts of SCM mice compared to controls, using stringent thresholds of adjusted P-value < 0.05 and |log_2_(fold change)| > 1 (Figure 5B).

Functional enrichment analyses were conducted on these DEGs derived from the mouse heart RNA-Seq data. Gene Ontology (GO) analysis of biological processes revealed significant enrichment of terms directly related to DDR, including "Double-Strand Break Repair" and "DNA Damage Response." Additionally, terms associated with inflammation ("Leukocyte Aggregation," "Chronic Inflammatory Response"), mitochondrial dynamics ("Regulation Of Mitochondrial Fission"), and various metabolic processes were enriched (Figure 5C). Correspondingly, GO cellular component analysis highlighted enrichment not only in "DNA Damage Foci" but also in mitochondrial structures ("Respiratory Chain Complex," "Oxidoreductase Complex") and cardiac muscle components ("Sarcoplasmic Reticulum," "Myofibril") (Figure 5D). Molecular function analysis indicated alterations in diverse activities, including "P53 Binding," "Antioxidant Activity," and metabolic substrate binding (Figure 5E). Kyoto Encyclopedia of Genes and Genomes (KEGG) pathway analysis further underscored metabolic disruption ("Tca Cycle," "Ppar pathway," "Cholesterol Metabolism"), stress responses ("Ferroptosis," "Hif-1 Signaling Pathway"), and inflammation-related pathways (Figure 5F).

To confirm the activation status of specific pathways relevant to DDR and sepsis within this LPS model, Gene Set Enrichment Analysis (GSEA) was performed. GSEA demonstrated significant positive enrichment in the SCM group for gene sets related to inflammatory responses ("Leukocyte aggregation," Figure 5G; "Chronic inflammatory response," Figure 5N). Importantly, strong enrichment was observed for multiple gene sets central to DDR, including "Double-strand break repair" (Figure 5H), "DNA damage response" (Figure 5J), "DNA repair-dependent chromatin remodeling" (Figure 5K), and "Nucleotide-excision repair" (Figure 5L). Furthermore, pathways associated with "DNA replication" (Figure 5M) and mitochondrial dynamics ("Regulation of mitochondrial fission," Figure 5I; "Mitochondrial fusion," Figure 5O) and were also significantly enriched, suggesting a complex interplay between DDR, mitochondrial stress, and cell cycle processes in the LPS-challenged heart.

In summary, transcriptomic analysis of heart tissue from a murine model of LPS-induced septic cardiomyopathy (GSE267388) reveals robust activation of DNA damage response and repair pathways. This DDR activation occurs concomitantly with pronounced inflammatory signatures and significant alterations in mitochondrial regulation and cellular metabolism, providing strong *in vivo* model evidence using RNA sequencing that supports the role of DNA damage in the cardiac pathophysiology of sepsis.

### Conserved DDR gene upregulation and pathway activation in sepsis are associated with poor prognosis

**Figure 6:**
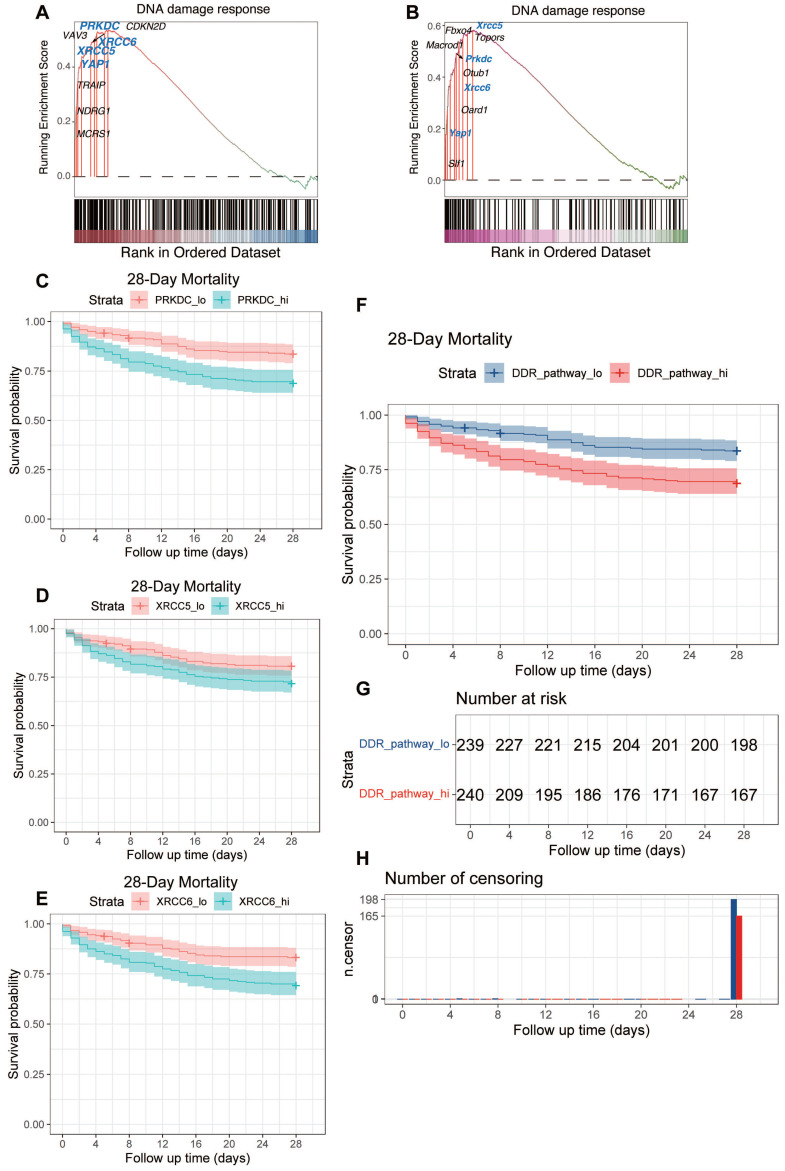
** Conserved DNA damage response activation predicts poor prognosis in human sepsis.** (A, B) Gene Set Enrichment Analysis (GSEA) plots illustrating the enrichment of the "DNA damage response" pathway in (A) human septic cardiomyopathy heart tissue (GSE79962) and (B) murine LPS-induced septic heart tissue (GSE267388). Key conserved upregulated genes identified within the leading-edge subset, including *YAP1*, *PRKDC*, *XRCC5*, and *XRCC6*, are highlighted, indicating cross-species relevance. (C-E) Prognostic significance analysis using the GSE65682 dataset, comprising whole blood transcriptomic data from human sepsis patients upon ICU admission linked to 28-day survival outcomes. Kaplan-Meier curves comparing 28-day survival between patients with high versus low expression levels of (C) *PRKDC*, (D) *XRCC5*, and (E) *XRCC6*. P-values were determined by log-rank test. (F-H) Patients were stratified into 'High' and 'Low' groups based on the median expression level or pathway activity score. (F) Kaplan-Meier curve comparing 28-day survival between patients with high versus low DNA Damage Response (DDR) pathway activity scores, calculated using single-sample GSEA (ssGSEA). P-value was determined by log-rank test. (G) Table showing the number of patients at risk over the 28-day follow-up period for the high and low DDR pathway activity groups depicted in panel F. (H) Bar chart comparing the absolute number of surviving patients at day 28 between the High-DDR and Low-DDR activity groups.

Following the identification of DNA Damage Response (DDR) pathway activation and the consistent upregulation of key DDR-associated genes, including *YAP1*, *PRKDC*, *XRCC5*, and *XRCC6*, across human and murine septic heart tissue datasets (GSE79962, Figure 6A; GSE267388, Figure 6B), we investigated the clinical relevance of these findings in human sepsis. We utilized the GSE65682 dataset, comprising whole blood transcriptomic data and 28-day survival outcomes from sepsis patients upon ICU admission.

We first examined the prognostic value of the conserved DDR genes identified earlier. Kaplan-Meier survival analyses based on gene expression levels in the GSE65682 cohort revealed that patients with higher expression of *PRKDC* had significantly poorer 28-day survival compared to those with lower expression (Figure 6C). Similarly, elevated expression of *XRCC5* (Figure 6D) and *XRCC6* (Figure 6E) was also significantly associated with increased 28-day mortality.

Next, we assessed the prognostic significance of the overall DDR pathway activity. The activity score for the DDR pathway was calculated for each patient using single-sample Gene Set Enrichment Analysis (ssGSEA). Patients were then stratified into 'High-DDR' and 'Low-DDR' activity groups based on the median score. Kaplan-Meier survival analysis demonstrated that the High-DDR activity group exhibited significantly worse 28-day survival compared to the Low-DDR activity group (Figure 6F. Figure 6G provides supplementary details for this survival analysis, showing the number of patients remaining at risk over the 28-day follow-up period in each group. Corroborating the survival curve analysis, a direct comparison of outcomes at the end of the follow-up period showed a significantly lower absolute number of surviving patients in the High-DDR activity group compared to the Low-DDR activity group (Figure 6H).

In summary, these analyses of the GSE65682 patient cohort demonstrate that higher expression levels of key DDR components (*PRKDC*, *XRCC5*, *XRCC6*) individually, as well as elevated overall DDR pathway activity (assessed via ssGSEA) in peripheral blood upon ICU admission, are significant indicators of poor 28-day prognosis in sepsis patients (Figure 6C-H).

### Single-cell analysis reveals upregulation of DDR genes PRKDC, XRCC5, and XRCC6 in specific cardiac cell populations during sepsis

To further investigate the cellular basis for the observed DNA Damage Response (DDR) activation in septic hearts, we performed an analysis of single-cell RNA sequencing (scRNA-seq) data from the GSE190856 dataset. This dataset profiles heart tissue from mice 3 days after cecal ligation and puncture (CLP)-induced sepsis (SCM model) and compares it to control (Con) mice (*Mus musculus*).

**Figure 7:**
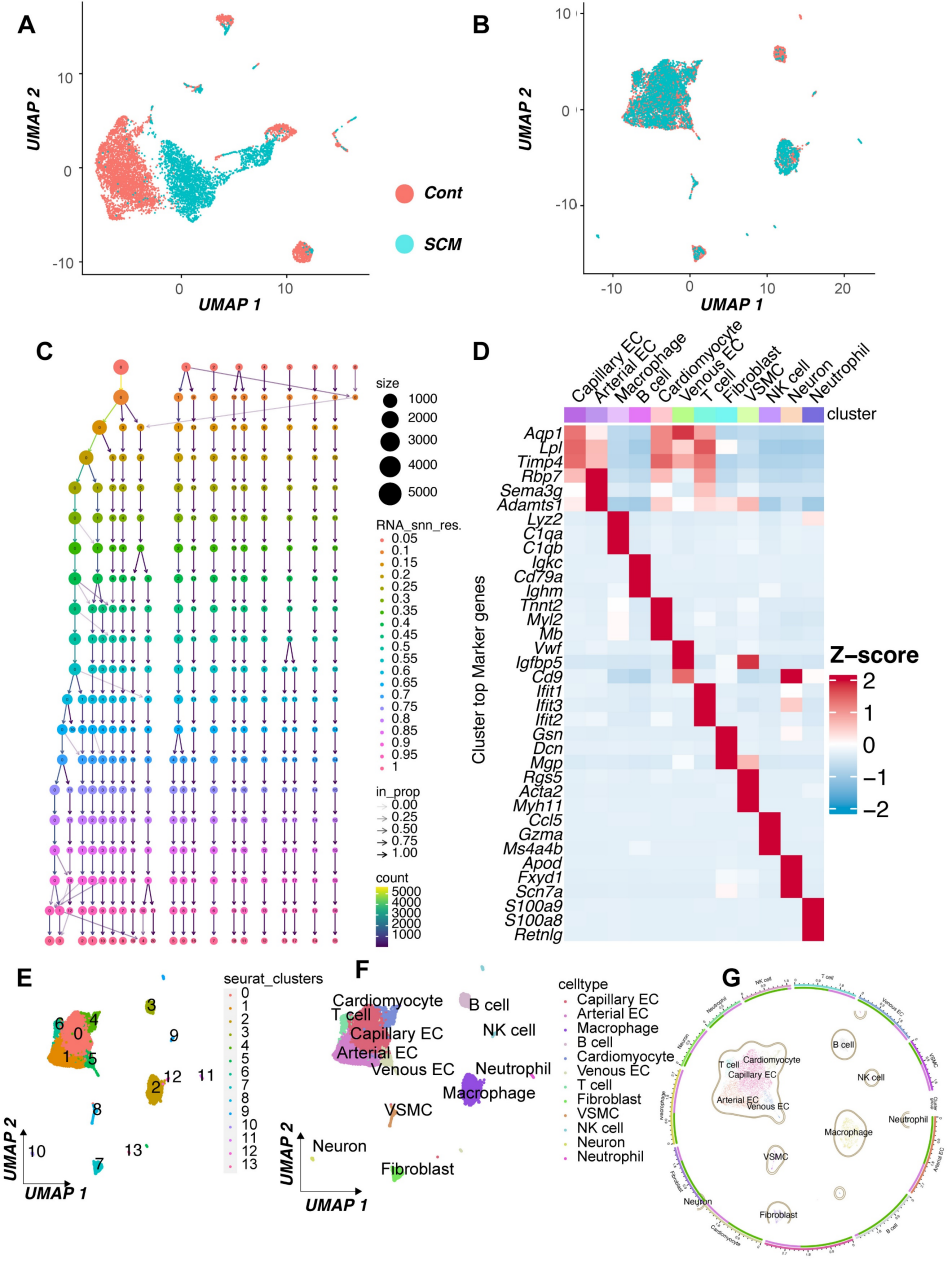

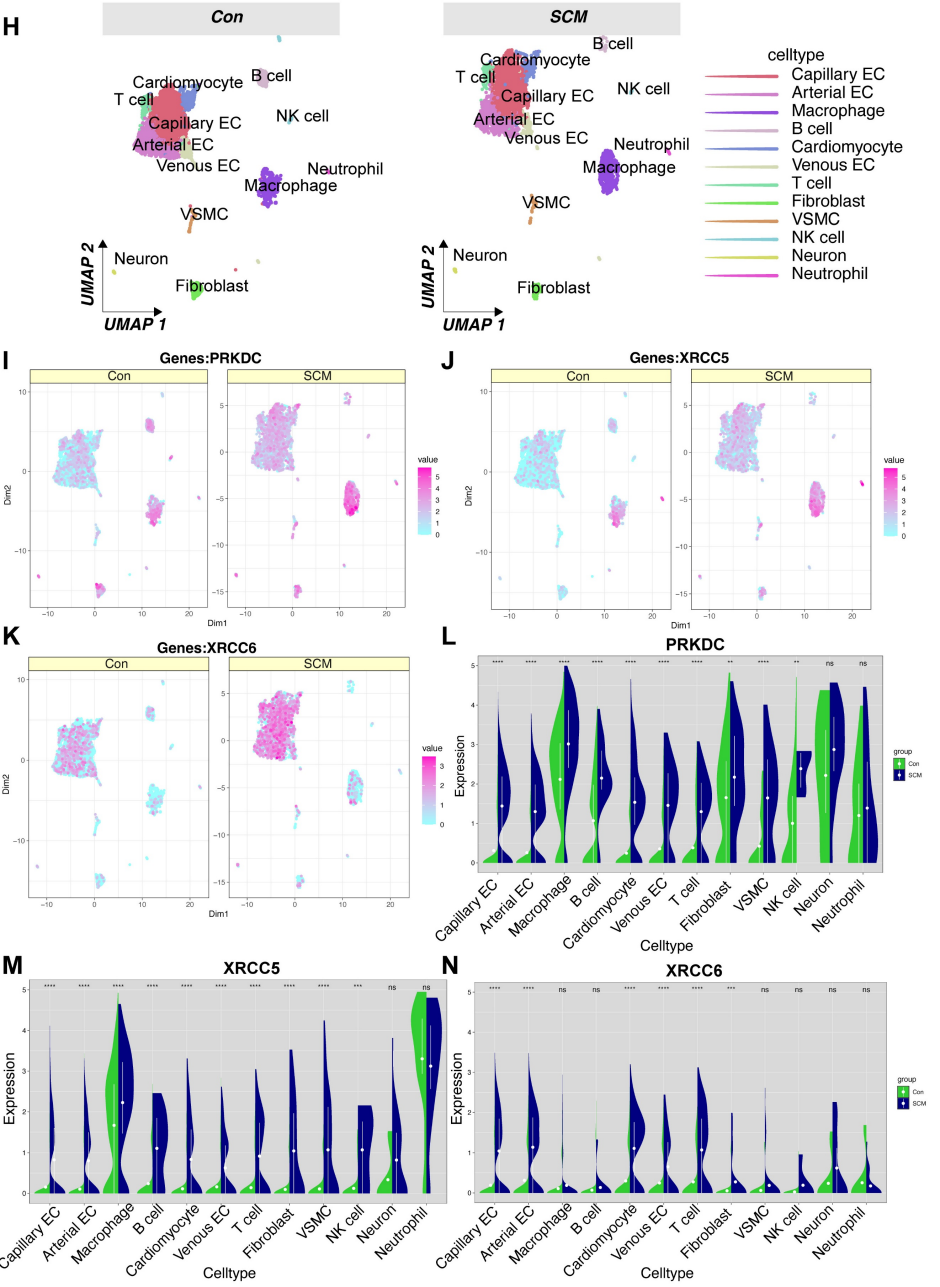
** Single-cell RNA sequencing analysis reveals cell-type specific upregulation of DDR genes in murine septic hearts.** Single-cell transcriptomic analysis of heart tissue isolated from control mice (Con) and mice 3 days post cecal ligation and puncture (CLP)-induced sepsis (SCM). (A, B) Uniform Manifold Approximation and Projection (UMAP) visualization of integrated single-cell transcriptomes, colored by experimental condition (e.g., A: Merged Con and SCM; B: Split view or alternative projection). (C, D) Identification of distinct cell clusters based on canonical marker gene expression, shown via (C) dot plot indicating the percentage of cells expressing marker genes (dot size) and average expression level (color intensity) per cluster, and (D) heatmap displaying scaled expression of top marker genes across clusters. (E, F) UMAP visualization colored by assigned cell type identity, identifying major cardiac populations including Cardiomyocytes (CM), Endothelial Cells (EC - Arterial, Capillary, Venous subtypes), Fibroblasts, Vascular Smooth Muscle Cells (VSMCs), Neurons, and various Immune cells (Macrophages, T cells, NK cells, Neutrophils). (G) Graphical summary shows the relative proportions of the identified cell types in the analyzed heart tissue. (H) UMAP visualizations split by experimental condition (Con vs. SCM), colored by cell type, facilitating comparison of cell population distributions and potential state changes induced by sepsis. (I-K) Feature plots overlaid on the UMAP demonstrating the expression levels and spatial distribution across cells for key DNA Damage Response (DDR) genes. Color intensity corresponds to the normalized expression level. (L-N) Violin plots comparing the expression distribution of DDR genes between SCM (e.g., purple violins) and Con (e.g., green violins) groups within major identified cell types: (L) *Prkdc*, (M) *Xrcc5*, and (N) *Xrcc6*. Height and width of violins represent density of cells at different expression levels.

Unsupervised clustering of the integrated single-cell transcriptomes projected using UMAP revealed distinct cell populations present in both control and septic hearts (Figure 7A-B). Based on the expression of canonical marker genes (Figure 7C-D), we identified major cardiac cell types including cardiomyocytes, fibroblasts, endothelial cells (ECs, further subdivided into arterial, capillary, and venous subtypes), vascular smooth muscle cells (VSMCs), neurons, and various immune cell populations such as macrophages, T cells, NK cells, and neutrophils (Figure 7E-F). A comparison of cell distributions suggested alterations in the cellular landscape following CLP, potentially reflecting inflammatory cell infiltration or changes in resident cell states (Figure 7G-H).

We specifically focused on the expression patterns of the key DDR genes *Prkdc*, *Xrcc5*, and *Xrcc6*, which were implicated in bulk tissue analyses and prognosis. Feature plots indicated that these genes were expressed across multiple cell types within the heart (Figure 7I-K), with visual inspection suggesting potentially broader or higher expression in the SCM condition.

To quantify cell-type-specific changes, we compared the expression distribution of these genes between the SCM (CLP day 3) and Con groups within each identified cell population using violin plots. This analysis revealed significant upregulation of *Prkdc* in the SCM group compared to controls, particularly notable in capillary ECs, fibroblasts, macrophages, and potentially cardiomyocytes (Figure 7L). Similarly, *Xrcc5* expression was significantly increased in the SCM condition across several cell types, including capillary ECs, venous ECs, fibroblasts, and potentially cardiomyocytes (Figure 7M). *Xrcc6* expression also demonstrated significant upregulation post-CLP, with pronounced increases observed in capillary ECs, macrophages, and potentially fibroblasts and VSMCs (Figure 7N).

These single-cell resolution data confirm the upregulation of crucial DDR components *Prkdc*, *Xrcc5*, and *Xrcc6* within the septic mouse heart 3 days post-CLP. Importantly, this analysis identifies specific cell populations contributing to this response, highlighting significant increases in endothelial cells (notably capillary ECs), fibroblasts, and infiltrating/resident immune cells, suggesting widespread DDR activation across both parenchymal and non-parenchymal cells in the septic myocardium.

### LPS induces mitochondrial fission and cytoskeleton remodeling pathways in endothelial cells

Given the observed activation of the DNA Damage Response (DDR) in endothelial cells upon septic stimuli, and the potential interplay between DDR, mitochondrial dynamics, and cellular structure, we investigated transcriptomic changes related to mitochondrial fission and cytoskeleton remodeling in human umbilical vein endothelial cells (HUVECs) following LPS exposure. We analyzed the publicly available microarray dataset GSE27912. This dataset profiled gene expression using the Affymetrix Human Gene 1.0 ST Array platform in primary HUVECs treated with lipopolysaccharide (LPS, specifically 100 ng/mL for 4 h) versus untreated control conditions.

**Figure 8:**
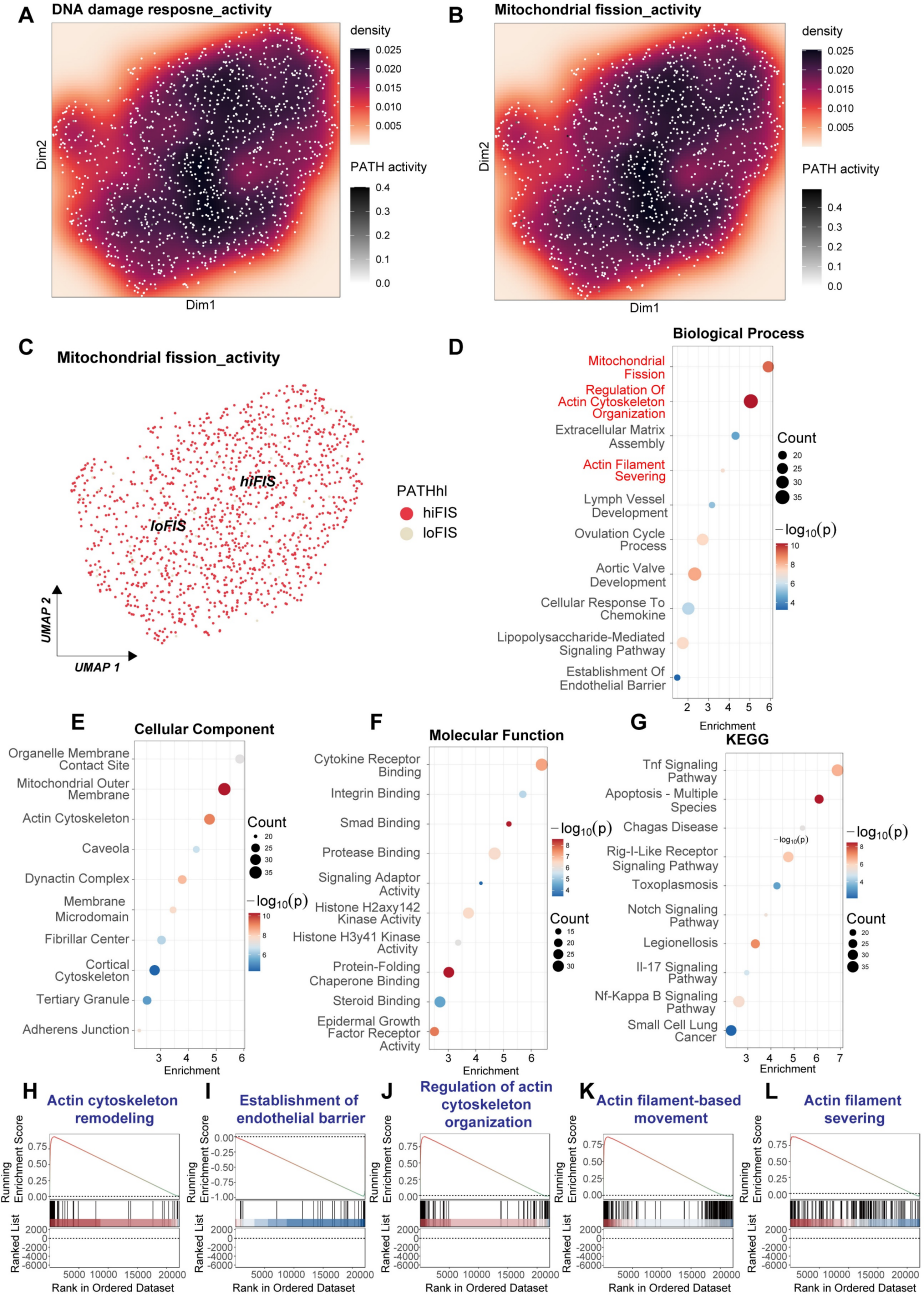
** Transcriptomic analysis reveals activation of mitochondrial fission and cytoskeleton remodeling pathways in LPS-stimulated HUVECs.** Bioinformatic analysis of microarray data derived from the GSE27912 dataset. This dataset profiled transcriptomes of Human Umbilical Vein Endothelial Cells (HUVECs) stimulated with lipopolysaccharide (LPS; 100 ng/mL for 4 h) compared to untreated control cells, using the Affymetrix Human Gene 1.0 ST Array platform. (A, B) Visualization of estimated pathway activity scores across samples, mapped onto dimension reduction coordinates (Dim1/Dim2). Density plots represent calculated activity for (A) DNA damage response and (B) Mitochondrial fission pathways. Color intensity correlates with pathway activity level. (C) UMAP projection potentially stratifying samples based on calculated mitochondrial fission activity scores (e.g., distinguishing high-fission [hiFIS] from low-fission [loFIS] samples). (D-G) Functional enrichment analysis performed on genes differentially expressed between LPS-treated and control HUVECs. Dot plots show significantly enriched terms for: (D) Gene Ontology (GO) Biological Process, highlighting terms related to mitochondrial fission, actin cytoskeleton organization, and endothelial barrier function. (E) GO Cellular Component, indicating involvement of mitochondrial membranes and cytoskeletal structures. (F) GO Molecular Function. (G) Kyoto Encyclopedia of Genes and Genomes (KEGG) pathways, confirming enrichment of inflammatory signaling pathways. In these plots, dot color typically represents the p-value, and dot size reflects the number of differentially expressed genes associated with the term. (H-L) Gene Set Enrichment Analysis (GSEA) plots illustrating the enrichment profiles for specific gene sets comparing LPS-treated versus control HUVECs. The plots show the running enrichment score (ES) across the ranked list of genes.

Analysis of this microarray data suggested potential co-activation or correlation between DDR and mitochondrial fission pathways upon LPS stimulation (Figure 8A-C). To systematically identify affected biological processes, we performed functional enrichment analysis on genes differentially expressed between the LPS-treated and control HUVECs. Gene Ontology (GO) analysis revealed significant enrichment in Biological Process terms directly related to mitochondrial dynamics and cytoskeleton architecture, including "Mitochondrial Fission", "Regulation Of Actin Cytoskeleton Organization", "Actin Filament Severing", and "Establishment Of Endothelial Barrier" (Figure 8D). Supporting these findings, enriched GO Cellular Component terms included "Mitochondrial Outer Membrane", "Actin Cytoskeleton", "Cortical Cytoskeleton", and "Adherens Junction" (Figure 8E). GO Molecular Function analysis indicated alterations in various binding and enzymatic activities (Figure 8F). Furthermore, Kyoto Encyclopedia of Genes and Genomes (KEGG) pathway analysis confirmed the expected enrichment of inflammatory signaling cascades induced by LPS, such as "Tnf Signaling Pathway" and "Nf-Kappa B Signaling Pathway" (Figure 8G).

To further validate the impact on cytoskeletal regulation within this experimental context, Gene Set Enrichment Analysis (GSEA) was conducted. GSEA demonstrated significant positive enrichment for multiple gene sets associated with actin dynamics and endothelial barrier function in the HUVECs treated with LPS for 4 h. These included "Actin cytoskeleton remodeling" (Figure 8H), "Establishment of endothelial barrier" (Figure 8I), "Regulation of actin cytoskeleton organization" (Figure 8J), "Actin filament-based movement" (Figure 8K), and "Actin filament severing" (Figure 8L). The positive enrichment scores indicate a coordinated upregulation of genes involved in these processes following LPS exposure.

In conclusion, the transcriptomic analysis of the GSE27912 dataset, derived from microarray profiling of HUVECs stimulated with LPS for 4 h, provides strong evidence for the activation of pathways governing mitochondrial fission and extensive remodeling of the actin cytoskeleton and endothelial barrier structures. These findings highlight the direct impact of LPS stimulation on crucial aspects of endothelial cell biology and support their potential connection to DDR activation.

### Heightened mitochondrial fission activity correlates with cytoskeletal dysregulation pathways in septic cardiac capillary endothelial cells

To investigate the molecular alterations underpinning sepsis-induced cardiomyopathy, focusing on the interplay between DNA Damage Response (DDR) sequelae like mitochondrial dynamics and cytoskeletal changes, we analyzed transcriptomic data derived from the GSE190856 study. This involved comparing gene expression profiles from whole heart tissue of mice subjected to CLP-induced sepsis (Day 3, denoted LPS in figure panels) against those from control mice (Con).

**Figure 9:**
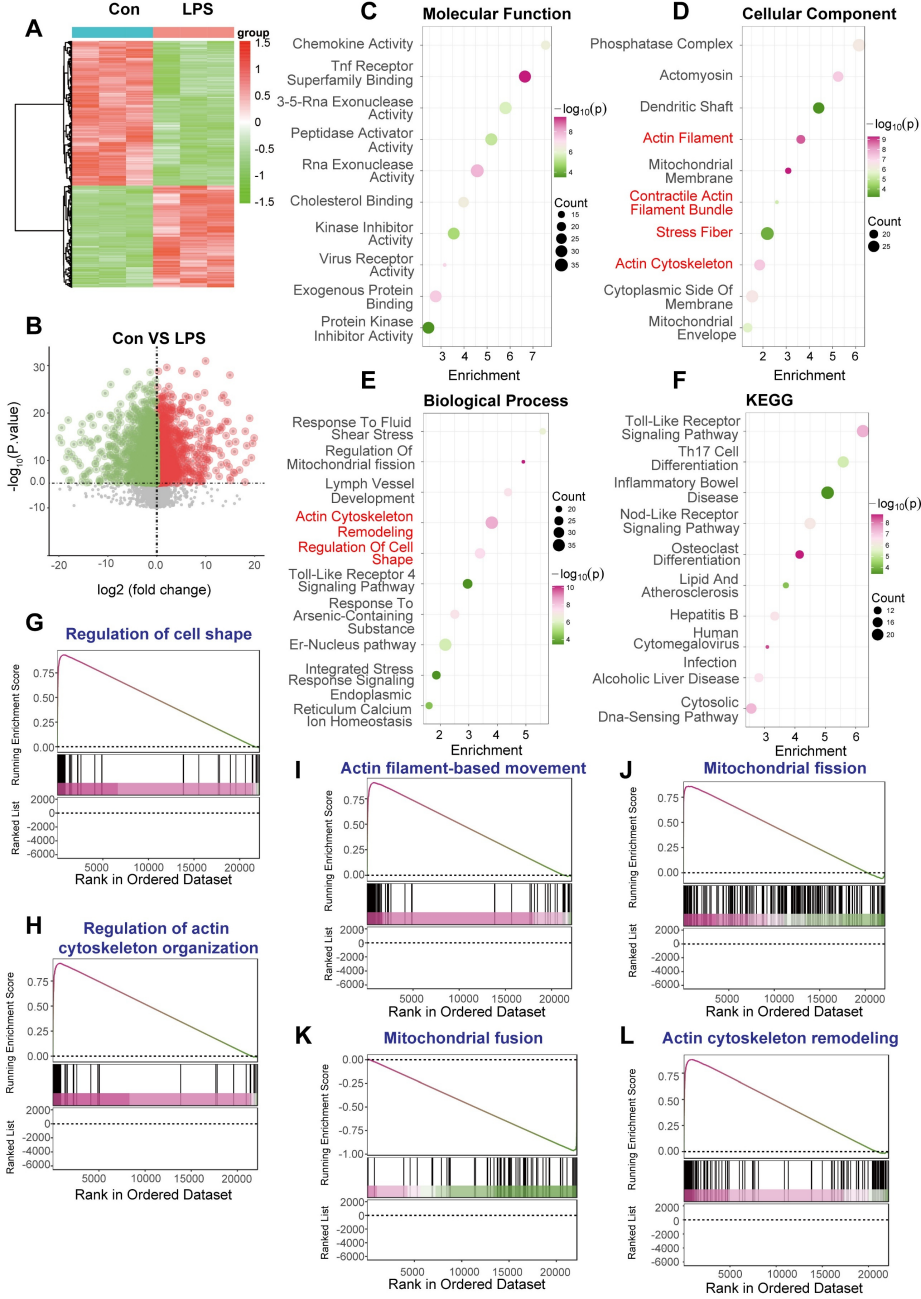
** Transcriptomic signatures link mitochondrial fission and cytoskeletal remodeling pathways in septic mouse hearts.** Bioinformatic analysis comparing gene expression profiles from whole heart tissue of mice subjected to CLP-induced sepsis (Day 3, labeled LPS) versus control mice (Con). (A) Heatmap displaying differentially expressed genes (DEGs) between the LPS and Con samples, illustrating distinct clustering patterns according to experimental condition. The color scale represents Z-score normalized expression levels. (B) Volcano plot visualizing differential gene expression. Red and green dots denote significantly upregulated and downregulated genes, respectively, in the LPS group compared to the Con group. (C-F) Functional enrichment analyses performed on the identified DEGs. (G-L) Gene Set Enrichment Analysis (GSEA) plots comparing the LPS versus Con groups for specific pathways. The plots display the running enrichment score (ES) across the ranked list of genes.

Significant differences in gene expression profiles were readily apparent between the septic (LPS) and control (Con) cardiac tissues. Unsupervised hierarchical clustering clearly segregated the samples based on experimental condition (Figure 9A). Volcano plot analysis further illustrated the extent of transcriptional perturbation, revealing numerous genes significantly up- and downregulated in the septic hearts compared to controls (Figure 9B).

Functional enrichment analyses were performed to elucidate the biological significance of these differentially expressed genes (DEGs). Gene Ontology (GO) analysis of enriched Molecular Function terms pointed towards altered signaling and binding activities (Figure 9C). More pertinently to our hypothesis, GO Cellular Component analysis demonstrated significant enrichment for terms related to both mitochondrial structure (“Mitochondrial Membrane”, “Mitochondrial Envelope”) and the actin cytoskeleton (“Actomyosin”, “Actin Filament”, “Contractile Actin Filament Bundle”, “Stress Fiber”, “Actin Cytoskeleton”) (Figure 9D). Consistently, GO Biological Process enrichment highlighted key processes including “Regulation Of Mitochondrial fission”, “Actin Cytoskeleton Remodeling”, and “Regulation Of Cell Shape”, alongside stress and inflammatory responses (“Response To Fluid Shear Stress”, “Toll-Like Receptor 4 Signaling Pathway”) (Figure 9E). Kyoto Encyclopedia of Genes and Genomes (KEGG) pathway analysis primarily revealed enrichment in inflammatory signaling cascades (“Toll-Like Receptor Signaling Pathway”, “Nod-Like Receptor Signaling Pathway”) and metabolic pathways (Figure 9F), reflecting the systemic nature of sepsis.

To further validate the activation status of pathways related to cytoskeletal dynamics and mitochondrial fission, Gene Set Enrichment Analysis (GSEA) was conducted. This analysis revealed significant positive enrichment in the septic (LPS) group for multiple gene sets governing cytoskeletal architecture, including “Regulation of cell shape' (Figure 9G), “Regulation of actin cytoskeleton organization” (Figure 9H), “Actin filament-based movement” (Figure 9I), and “Actin cytoskeleton remodeling” (Figure 9L). Crucially, GSEA also demonstrated significant positive enrichment for “Mitochondrial fission” (Figure 9J) and concomitant negative enrichment (downregulation) for “Mitochondrial fusion” (Figure 9K), strongly indicating a shift towards mitochondrial fragmentation in the septic heart.

In conclusion, transcriptomic analysis of cardiac tissue from this CLP-induced murine sepsis model demonstrates profound gene expression changes. Functional analyses robustly highlight the concurrent activation of pathways driving mitochondrial fission and extensive remodeling of the actin cytoskeleton, alongside inflammatory responses. These molecular signatures strongly support the involvement of perturbed mitochondrial dynamics and cytoskeletal integrity as key pathological features in sepsis-induced cardiomyopathy.

### Pharmacological inhibition of DNA-PKcs attenuates LPS-induced cardiamyopathy, mitochondrial fragmentation, and cytoskeletal disarray in vivo

To empirically substantiate our bioinformatic inferences regarding the role of the DNA Damage Response (DDR) in sepsis-associated organ injury, we employed an *in vivo* murine model of lipopolysaccharide (LPS)-induced sepsis. We systematically evaluated the impact of LPS challenge, and the potential therapeutic efficacy of concurrent DDR inhibition via the DNA-PKcs specific inhibitor NU7441, on cardiac function, microvascular integrity, mitochondrial morphology, and cytoskeletal architecture.

**Figure 10:**
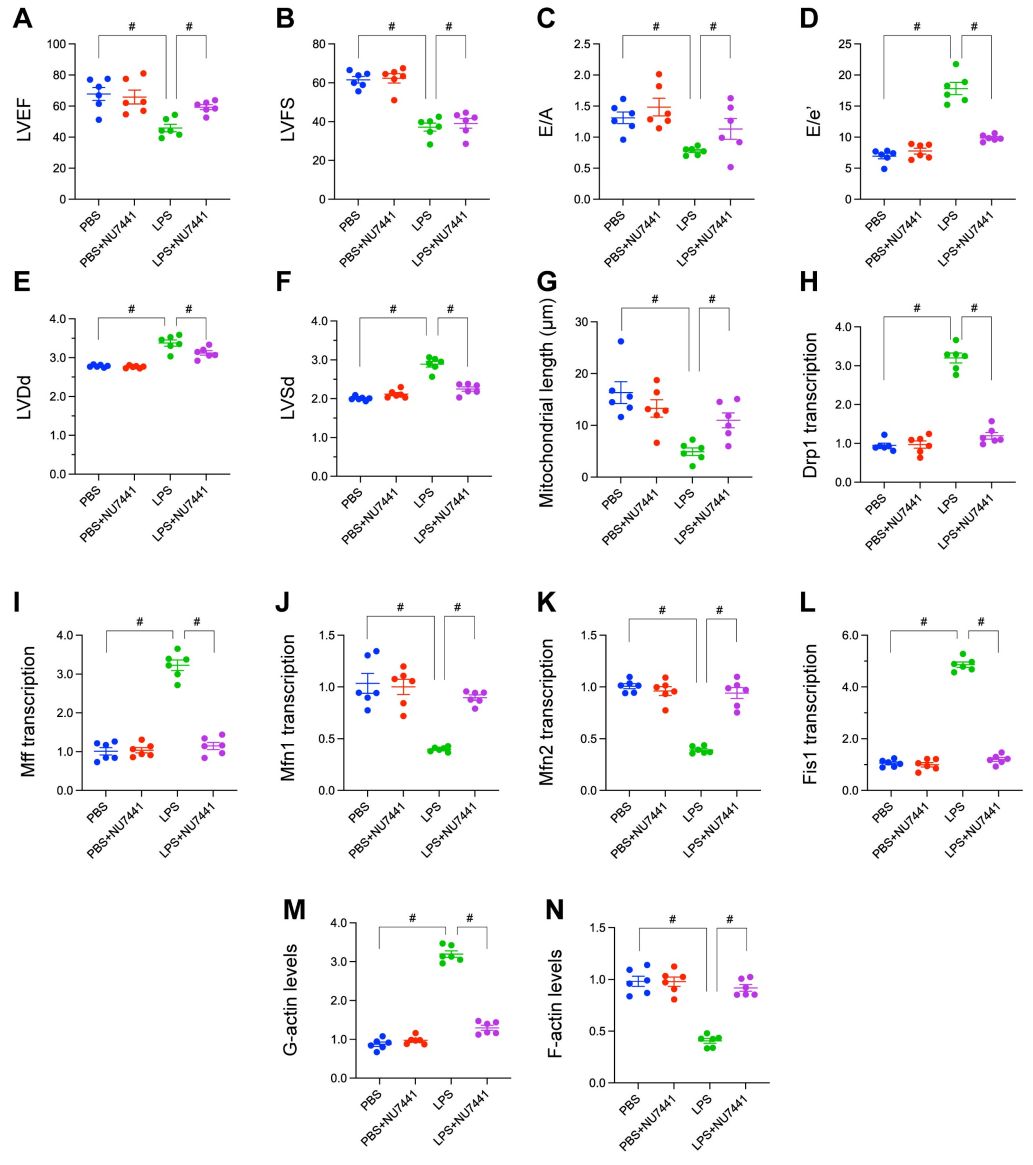
** Inhibition of DNA damage response ameliorates LPS-induced cardiomyopathy, mitochondrial fission, microvascular injury, and cytoskeletal disruption.** *In vivo* validation experiments using a murine model of LPS-induced sepsis, comparing PBS (Control), PBS + NU7441 (DNA-PKcs inhibitor control), LPS, and LPS + NU7441 treatment groups. (A-F) Echocardiographic assessment of cardiac function. Quantification of (A) Left Ventricular Ejection Fraction (LVEF, %), (B) Left Ventricular Fractional Shortening (LVFS, %), (C) E/A ratio, (D) E/e' ratio, (E) Left Ventricular End-Diastolic Dimension (LVDd, mm), and (F) Left Ventricular End-Systolic Dimension (LVSd, mm). (G) Representative micrographs of Hematoxylin and Eosin (HE) stained cardiac tissue sections, illustrating microvascular morphology in the different treatment groups. Scale bar = 75 µm. (H) Quantification of average mitochondrial length (µm) derived from transmission electron microscopy images of cardiomyocytes. (I-L) Relative mRNA expression levels of mitochondrial dynamics regulators quantified by qRT-PCR: (J) *Drp1*, (K) *Mff*, (L) *Mfn1*, (M) *Mfn2*, and (N) *Fis1*. (M-N) Quantification of relative levels of (M) globular actin (G-actin) and (N) filamentous actin (F-actin). Data are presented as mean ± SD. #P < 0.05.

Comprehensive echocardiographic assessments were conducted to delineate alterations in cardiac performance. Exposure to LPS precipitated pronounced systolic dysfunction, evidenced by significant reductions in Left Ventricular Ejection Fraction (LVEF; Figure 10A) and Fractional Shortening (LVFS; Figure 10B) relative to vehicle-treated controls. Diastolic function was similarly compromised following LPS challenge, characterized by a significant decrease in the E/A ratio, suggesting impaired ventricular relaxation (Figure 10C), and a marked elevation in the E/E' ratio, indicative of increased left ventricular filling pressures (Figure 10D). Furthermore, LPS induced adverse ventricular remodeling, manifest as significant increases in Left Ventricular End-Diastolic Dimension (LVDd; Figure 10E) and End-Systolic Dimension (LVSd; Figure 10F). Critically, concurrent administration of NU7441 with LPS significantly attenuated these multifaceted detrimental effects on cardiac function, preserving both systolic contractility and diastolic relaxation while mitigating ventricular dilation.

Ultrastructural analysis via transmission electron microscopy enabled quantification of mitochondrial morphology. Cardiomyocytes from LPS-treated mice exhibited a significant reduction in average mitochondrial length compared to controls, consistent with augmented mitochondrial fission. Administration of NU7441 effectively abrogated this LPS-mediated mitochondrial fragmentation, maintaining mitochondrial lengths comparable to baseline (Figure 10G).

To elucidate the molecular mechanisms governing these mitochondrial dynamic changes, the transcription of key regulatory genes was quantified. LPS administration resulted in a significant upregulation of transcripts encoding the pro-fission proteins *Drp1*, *Mff*, and *Fis1* (Figure 10H-L). Conversely, LPS concomitantly downregulated the expression of transcripts encoding the pro-fusion proteins *Mfn1* and *Mfn2* (Figure 10H-L). This transcriptional reprogramming favoring mitochondrial fission was largely normalized by DDR inhibition with NU7441.

Finally, the integrity of the actin cytoskeleton was evaluated. Qualitative imaging suggested conspicuous disruption and disorganization of filamentous actin (F-actin) structures within cardiac tissue sections from LPS-treated mice, a phenotype partially preserved by NU7441 (Figure 10M-N). Quantitative biochemical analysis further corroborated this observation, revealing a significant increase in the pool of globular actin (G-actin; Figure 10M-N) and a corresponding decrease in F-actin levels (Figure 10M-N) in the LPS group, signifying enhanced actin depolymerization. NU7441 administration significantly attenuated these LPS-induced shifts in the actin polymerization equilibrium.

In aggregate, these *in vivo* validation studies rigorously demonstrate that LPS challenge precipitates a complex pathophysiology encompassing impaired cardiac mechanics, mitochondrial fragmentation, microvascular injury associated with eNOS depletion, transcriptional skewing towards mitochondrial fission, and profound actin cytoskeletal disarray. The substantial amelioration of this constellation of pathologies by the DNA-PKcs inhibitor NU7441 strongly implicates DDR activation as a pivotal upstream orchestrator of sepsis-induced cardiac and microvascular injury.

### Pharmacological inhibition of mitochondrial fission ameliorates LPS-induced cardiomyopathy and microvascular injury

To directly investigate the functional role of mitochondrial fission in the pathogenesis of sepsis-induced cardiac and microvascular injury, we employed Mdivi-1, a pharmacological inhibitor targeting the key fission mediator Drp1, within the established lipopolysaccharide (LPS)-induced murine sepsis model. The capacity of Mdivi-1 to mitigate LPS-elicited pathologies was systematically evaluated through assessments of cardiac function and microvascular integrity.

**Figure 11:**
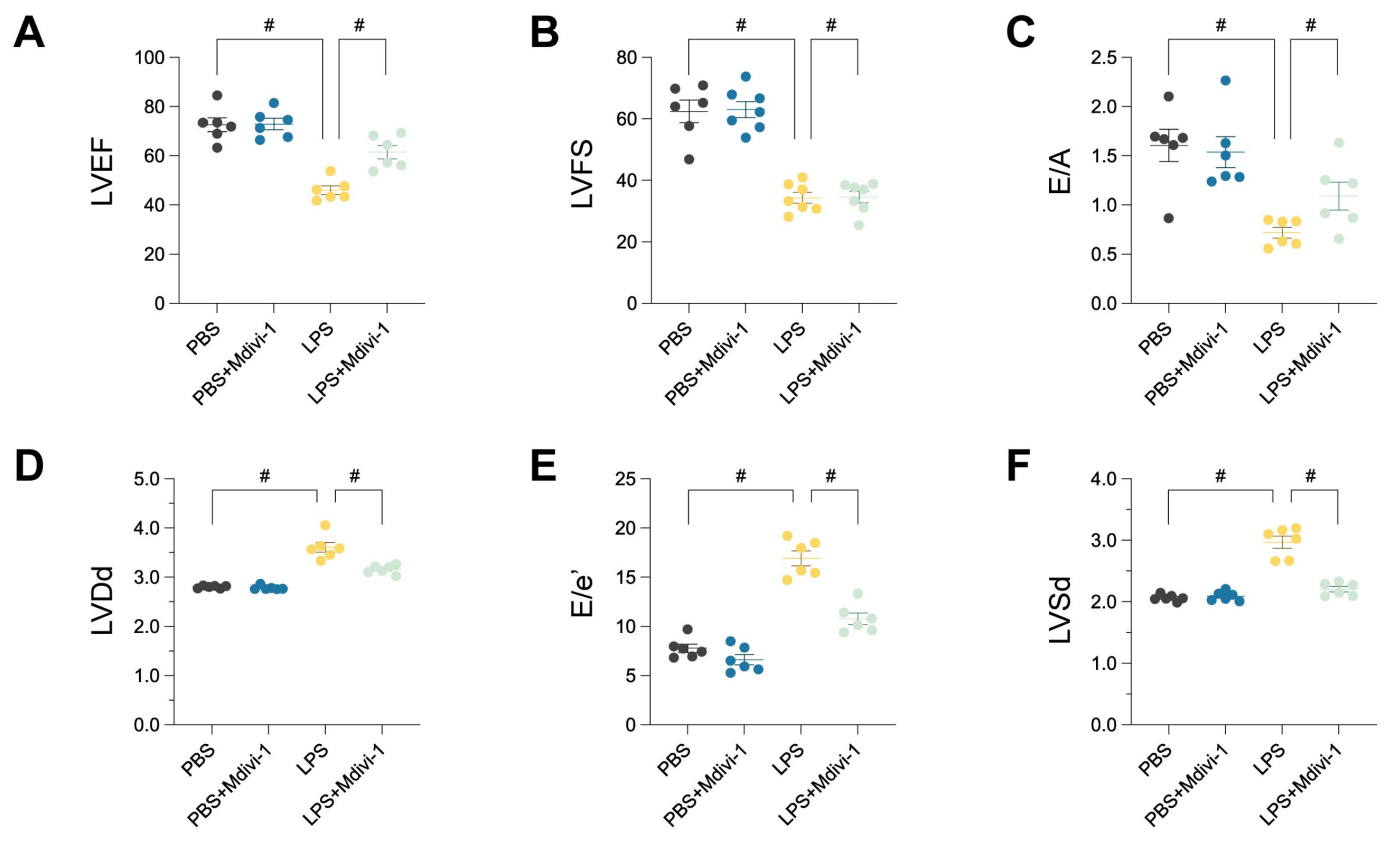
** Pharmacological inhibition of mitochondrial fission mitigates LPS-induced cardiac dysfunction and microvascular injury.** *In vivo* validation experiments using a murine model of LPS-induced sepsis, comparing PBS (Control), PBS + Mdivi-1 (mitochondrial fission inhibitor control), LPS, and LPS + Mdivi-1 treatment groups. (A-F) Echocardiographic assessment of cardiac function. Quantification of (A) Left Ventricular Ejection Fraction (LVEF, %), (B) Left Ventricular Fractional Shortening (LVFS, %), (C) E/A ratio, (D) Left Ventricular End-Diastolic Dimension (LVDd, mm), (E) E/e' ratio, and (F) Left Ventricular End-Systolic Dimension (LVSd, mm). #P < 0.05.

Comprehensive echocardiographic analyses were performed to characterize cardiac performance. As anticipated, LPS administration precipitated severe systolic dysfunction, evidenced by substantial reductions in Left Ventricular Ejection Fraction (LVEF; Figure 11A) and Left Ventricular Fractional Shortening (LVFS; Figure 11B) compared to vehicle-treated controls. Diastolic function was also significantly impaired, reflected by a marked decrease in the E/A ratio (Figure 11C). Furthermore, LPS challenge induced significant left ventricular dilation, as indicated by increased Left Ventricular End-Diastolic Dimension (LVDd; Figure 11D). Concomitantly, the E/e' ratio, a gauge of left ventricular filling pressures, was significantly elevated in the LPS group (Figure 11E). Left Ventricular End-Systolic Dimension (LVSd) was also significantly increased, consistent with impaired systolic emptying (Figure 11F). Critically, concurrent treatment with Mdivi-1 provided significant cardioprotection, markedly attenuating the LPS-induced decrements in LVEF and LVFS, normalizing diastolic parameters (E/A and E/e'), and preventing adverse ventricular remodeling as evidenced by the mitigation of LVDd and LVSd increases.

Histological examination of cardiac tissue sections stained with Hematoxylin and Eosin (HE) was conducted to assess microvascular structural integrity. Myocardial sections from LPS-treated animals displayed evidence of microvascular injury and architectural disturbances. In contrast, the microvasculature in animals receiving LPS concurrently with Mdivi-1 appeared largely preserved, suggesting that inhibition of mitochondrial fission protects against sepsis-associated vascular damage.

To further probe endothelial status, immunohistochemical analysis for endothelial nitric oxide synthase (eNOS) was performed. A pronounced reduction in eNOS immunoreactivity within the cardiac microvascular endothelium was observed following LPS challenge, indicative of endothelial dysfunction. This LPS-mediated downregulation of eNOS was substantially reversed by co-administration of Mdivi-1, suggesting restoration of endothelial functional marker expression through fission inhibition.

In aggregate, these experiments demonstrate that pharmacological inhibition of mitochondrial fission via Mdivi-1 significantly ameliorates LPS-induced cardiac dysfunction, encompassing both systolic and diastolic impairments, and mitigates associated ventricular dilation. Moreover, Mdivi-1 treatment preserved microvascular structural integrity and partially restored endothelial eNOS expression. These findings strongly implicate excessive mitochondrial fission as a key pathogenic mechanism downstream of LPS stimulation that contributes directly to the development of sepsis-induced cardiomyopathy and microvascular endothelial injury.

## Discussion

Sepsis is a severe medical condition characterized by a systemic inflammatory response to infection, often leading to multiorgan dysfunction, including cardiac complications. The cardiac microvasculature plays a crucial role in sepsis-induced cardiac dysfunction, as it is responsible for regulating blood flow and oxygen delivery to the myocardium. The interplay between sepsis and cardiac microvascular dysfunction involves complex mechanisms, including endothelial dysfunction, microvascular perfusion heterogeneity, and inflammatory responses. This answer explores the pathophysiology of cardiac microvascular dysfunction in sepsis, highlighting key mechanisms and potential therapeutic targets.

Sepsis-induced cardiac dysfunction is associated with impaired myocardial circulation due to microvascular dysfunction and ventriculo-arterial uncoupling 90, 91. This results in inadequate oxygen delivery to cardiac tissues, contributing to myocardial depression. Sepsis can lead to a mismatch between macrocirculation and microcirculation, resulting in heterogeneous blood flow distribution. This heterogeneity can induce a hyperdynamic circulation with high cardiac output, as demonstrated by mathematical models that show increased total flow when microvascular perfusion is uneven 92. Endothelial cells in the cardiac microvasculature are crucial for maintaining vascular tone and barrier function. In sepsis, endothelial dysfunction is exacerbated by factors such as DNA-PKcs-mediated phosphorylation of cofilin2, leading to F-actin depolymerization and impaired endothelial barrier integrity 93. This dysfunction contributes to increased vascular permeability and inflammation. The production of ROS and inflammatory cytokines in the endothelium leads to further microvascular damage and impaired myocardial oxygen consumption 94. This oxidative stress and inflammation can perpetuate a cycle of endothelial damage and microvascular dysfunction. Ulinastatin, a protease inhibitor, has been shown to protect against LPS-induced cardiac microvascular endothelial cell dysfunction by downregulating lncRNA MALAT1 and EZH2, reducing cell permeability and apoptosis 95. This suggests potential therapeutic strategies targeting specific molecular pathways to improve microvascular function. While the focus is often on the microvascular dysfunction in sepsis, it is important to consider the broader context of systemic inflammation and its impact on other organ systems. The interplay between systemic and local factors can complicate the clinical picture, necessitating a comprehensive approach to treatment. Understanding the specific mechanisms of cardiac microvascular dysfunction in sepsis can inform targeted interventions, potentially improving outcomes for patients with sepsis-induced cardiac complications.

The relationship between DNA damage response (DDR) and lipopolysaccharide (LPS) is multifaceted, involving both direct and indirect interactions that influence cellular and immune responses. LPS, a component of the outer membrane of Gram-negative bacteria, is known to trigger inflammatory responses and can also induce DNA damage 96, 97. This interaction is significant in understanding how inflammation and immune responses are modulated in the presence of bacterial infections. LPS can induce DNA damage in various cell types 42, 98-101, including sensory neurons and intestinal cells. This damage is often mediated by the production of reactive oxygen and nitrogen species (ROS/RNS) during inflammation, which can alter neuronal sensitivity and contribute to chronic inflammatory pain 102, 103. In intestinal cells, LPS from Helicobacter species has been shown to interfere with DNA repair mechanisms, increasing the risk of genotoxic effects and potentially contributing to conditions like inflammatory bowel disease and colon cancer 104, 105. LPS exposure can lead to epigenetic alterations in mitochondrial DNA, affecting the inflammatory response. For instance, LPS stimulation in human cells resulted in decreased mitochondrial DNA methylation, which correlated with changes in cytokine expression, suggesting a role for demethylated mtDNA in regulating immune responses 106, 107. The DNA damage response (DDR) is crucial for maintaining genomic integrity and involves several pathways that detect and repair DNA lesions 108-111. In the context of LPS exposure, enhancing DNA repair mechanisms, such as through the enzyme APE1, can mitigate DNA damage and its effects on neuronal function 102, 112. The DDR is not only a repair mechanism but also plays a role in immune defense. It has been shown that DDR proteins can activate innate immune responses upon detecting cytosolic pathological nucleic acids, indicating a crosstalk between DDR and immune pathways 113-115. The interplay between LPS-induced DNA damage and the DDR has significant implications for understanding inflammatory diseases and developing therapeutic strategies 116, 117. While LPS can exacerbate DNA damage and inflammation, enhancing DNA repair pathways offers a potential avenue for mitigating these effects. Moreover, the crosstalk between DDR and immune responses underscores the complexity of cellular responses to bacterial infections and the potential for targeting these pathways in disease treatment 118, 119. Understanding these interactions is crucial for developing interventions that can modulate immune responses and improve outcomes in conditions associated with chronic inflammation and DNA damage.

While this study provides compelling evidence linking LPS-induced DNA Damage Response (DDR), DNA-PKcs activation, mitochondrial fission, and actin disruption to cardiac microvascular dysfunction, several limitations should be acknowledged. Firstly, the study heavily relies on bioinformatic analyses of publicly available datasets. While powerful for hypothesis generation and demonstrating correlations across different models and species, these datasets originate from various laboratories using potentially different protocols, platforms (microarray, RNA-seq, scRNA-seq), time points, and specific model variations (LPS dose, cell types, clinical definitions of sepsis) 108, 120, 121. This inherent heterogeneity could introduce confounding factors or batch effects, although efforts like Harmony integration were used for scRNA-seq. Furthermore, bioinformatic findings, such as pathway enrichment (GSEA, ssGSEA) and correlation analyses (e.g., between DDR and fission/cytoskeleton scores in scRNA-seq) 122, 123, primarily establish associations rather than definitive causal links. Secondly, the *in vivo* validation predominantly used a murine model of endotoxemia induced by a single high dose of LPS (10 mg/kg). While this model effectively induces cardiac dysfunction and microvascular injury, it may not fully replicate the complex pathophysiology of human clinical sepsis, which is often polymicrobial, progresses over different time courses, and occurs in patients with various comorbidities. Findings derived from this specific LPS model should be cautiously extrapolated to the broader context of human sepsis 124-126. Additionally, most *in vivo* experiments assessed outcomes at a single 24 h time point, providing a snapshot rather than a dynamic view of the pathological processes. Thirdly, the study relies on pharmacological inhibitors, NU7441 for DNA-PKcs and Mdivi-1 for mitochondrial fission (Drp1) 127-130. Although these are widely used tools, the possibility of off-target effects contributing to the observed phenotypes cannot be entirely ruled out, especially in a complex *in vivo* setting. The study lacks complementary genetic validation using knockout or conditional knockout models for DNA-PKcs or Drp1 to definitively confirm the specificity of the observed inhibitor effects 131-134. Fourthly, while the study effectively links DNA-PKcs activation to downstream mitochondrial and cytoskeletal changes through inhibitor studies, the precise molecular mechanisms connecting these events require further elucidation. Finally, the reliance on transcriptomic data (mRNA levels) as a primary readout, while informative, may not always directly reflect protein expression levels or functional activity, which are often the ultimate mediators of cellular effects. Integrating proteomic or phosphoproteomic analyses could strengthen the mechanistic conclusions.
